# A Roadmap for Plasma‐Enabled Electrocatalysis in Urea Production

**DOI:** 10.1002/adma.202509784

**Published:** 2025-08-13

**Authors:** Jingwen Huang, Zhongping Qu, Renwu Zhou, Jing Sun, Rusen Zhou, Dorna Esrafilzadeh, Ali R. Jalili

**Affiliations:** ^1^ State Key Laboratory of Electrical Insulation and Power Equipment Centre for Plasma Biomedicine Xi'an Jiaotong University Xi'an 710049 P. R. China; ^2^ State Grid Suzhou Power Supply Company Suzhou Jiangsu 210000 P. R. China; ^3^ Graduate School of Biomedical Engineering University of New South Wales (UNSW) Sydney NSW 2052 Australia; ^4^ School of Chemistry University of New South Wales (UNSW) Sydney NSW 2052 Australia

**Keywords:** electrocatalysis, nitrogen fixation, plasma oxidation, techno‐economics, urea synthesis

## Abstract

Conventional Haber–Bosch/Bosch–Meiser routes link global urea production to fossil fuel‐based ammonia, accounting for ≈2% of the world's energy use and ≈1.5% of CO_2_ emissions. A modular, fully electrified alternative is charted that cleaves the problem at its natural fault line: a non‐thermal plasma first upgrades air to nitrate, then a CO_2_/NO_3_^−^ co‐electrolyzer stitches the two C─N bonds of urea at ambient conditions. The lens is deliberately cross‐disciplinary: every bottleneck is probed with the question, “Has a cognate field already cracked this?” If so, how can the solution be mirrored here? Plasma physics contributes to vibrational pumping, power modulated reactors, and in water quenching; CO_2_ and nitrate electro‐reduction supply relay‐site catalyst design, vacancy tuning, and pulsed‐bias choreography; flow‐battery engineering guides carbonate‐resilient gas‐diffusion electrodes (GDEs) and zero‐gap membrane‐electrode assemblies (MEAs); and analytical chemistry adds two‐probe assays that unmask false‐positive amine/amide signals. Stitching these advances together, techno‐economic modeling shows that sub‐megajoule plasmas, ≥70% urea‐selective in the electrolyzer, and renewable electricity (RE) at ≤3.5¢ kWh^−1^ can push green urea below the fossil‐based benchmark.

## Graphical abstract

A modular plasma—electrochemical platform enables fully electrified urea synthesis from air and CO_2_ under ambient conditions. This Review integrates plasma oxidation, C─N coupling electrolysis, mechanistic insights, and techno‐economic metrics into a roadmap for scalable, distributed, and renewable fertilizer production.

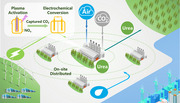

## 1 Introduction

Urea synthesis is situated at the intersection of two significant chemical challenges: the cost‐effective utilization of carbon and the sustainable activation of nitrogen.^[^ ^1^ ^]^ Historically, we have solved neither without significant fossil fuel inputs. The Haber–Bosch and Bosch–Meiser sequences cleave the N≡N bond at 150–250 bar and 400–500 °C, producing ammonia that is then converted to urea with CO_2_ at similarly harsh conditions. The energy penalty is enormous, the plants are massive and geographically immobile, and the entire chain is unsuitable for intermittent wind and solar power generation.^[^ ^2^ ^]^ Reinventing this chemistry for an electrified, modular age begins with the most direct thought experiment: could we skip ammonia entirely and forge the C─N bond electrochemically from N_2_ and CO_2_, two abundant but stubbornly inert gases?

That question has been the focus of researchers worldwide for the past decade. Sophisticated molecular catalysts, copper‐based nanostructures, and heteroatom‐doped carbons have been designed to simultaneously bind *CO_x_ and incipient *N_2_ fragments under mild cathodic potentials. Operando vibrational spectroscopy and density‐functional theory have revealed increasingly detailed information about surface chemistry, while GDEs and zero‐gap flow cells are now pushing current densities into the tens of milliamps per square centimeter.^[^ ^1a^ ^]^ Nonetheless, even on the most advanced materials, faradic efficiencies for urea rarely exceed 10%, total C─N yields hover in the micromole‐per‐hour range, and product‐specific energy remains orders of magnitude higher than any credible economic target.^[^ ^3^ ^]^ Three interconnected physicochemical realities explain the impasse. Direct electro‐reduction of N_2_ is sluggish due to the eight‐electron pathway competing with the two‐electron hydrogen‐evolution reaction, resulting in most electrons escaping as H_2_. Secondly, even when *N‐ and *C‐containing intermediates form, they almost always desorb independently (*NH_x_ reducing to NH_3_, *CO_x_ evolving to CO or formate) before the kinetically demanding C─N coupling step can occur. Third, both reactants are poorly soluble in water; steep concentration gradients, carbonate precipitation, and electrolyte flooding all work together to reduce the likelihood that the rare *NH_x_ and *CO moieties ever come into contact on a catalyst surface.^[^ ^4^ ^]^ It is an industrial non‐starter but an excellent intellectual playground.

Recognizing these challenges, the scientific community has shifted its focus to employ more reactive nitrogen vectors, such as nitrate, nitrite, and nitric oxide, eliminating the need to break N≡N at the interface. These oxy‐anions transport nitrogen in an oxidation state (+5 to +1) much closer to urea's (−3), dissolve at molar concentrations, and accept electrons via well‐mapped multi‐step cascades. Combining nitrate and CO_2_ reduction in a single electrolyzer is the most environmentally friendly and feasible option for replacing Bosch–Meiser.^[^ ^5^ ^]^ In practice, new complications arise. The catalyst hosts *CO/*CHO and *NO_2_/*NH_2_ intermediates simultaneously while rejecting side channels to NH_3_ and CO due to the sixteen‐electron, eighteen‐proton dance from NO_3_^−^ + CO_2_ to CO(NH_2_)_2_. CO_2_ acidifies the boundary layer, leading to acute proton management, while nitrate reduction prefers neutral or mildly alkaline media. Finally, nitrate must come from somewhere; the decarbonization narrative collapses if it is mined from nitric‐acid plants that still use natural gas.^[^ ^3^ ^]^

At this point, non‐thermal plasma provides a means of closing the loop.^[^ ^6^ ^]^ In our laboratory, we have shown that room‐temperature electrical discharges, whether dielectric barrier discharge (DBD), gliding arc or spark discharges driven by alternating current (AC), direct current (DC) or nanosecond pulse, directly convert air to a cocktail of NO, NO_2_ and other high‐valence NO_x_, which, when bubbled into water, yields nitrate and nitrite at specific energies below 3 kWh mol^−^^1^ of N‐oxide.^[^ ^7^ ^]^ We have already demonstrated that feeding this plasma‐derived nitrate to a copper‐based cathode yields ammonia at respectable current densities and FEs approaching ≈100%.^[^ ^8^ ^]^ For urea, the conceptual leap is straightforward: maintain the plasma module as an electricity‐to‐nitrate generator, but pair it with a C─N coupling cathode in a CO_2_‐saturated flow cell, rather than an NH_3_‐selective cathode. The nitrate reservoir acts as a chemical buffer between two reactors and two rate regimes, smoothing the temporal fluctuations inherent in renewable power and decoupling the harsh kinetics of N_2_ activation from the subtle dance of C─N assembly.

This hybrid approach transforms each of the existing bottlenecks. The slow, electron‐hungry breaking of N≡N is offloaded to the plasma, where energetic electrons, not the catalyst surface, perform the activation. The electrocatalyst begins its work from nitrate, a soluble species with only four electrons shy of the *NH_2_ moiety needed for coupling.^[^ ^9^ ^]^ Bimetallic or dual‐site catalysts can adjust binding energies for *CO and *NH_2_ without accommodating *N_2_, addressing surface selectivity through rational design.^[^ ^10^ ^]^ Local pH and electrolyte composition can be optimized independently of plasma discharge physics, eliminating the CO_2_/nitrate incompatibility in one‐pot cells. Even analytics become cleaner as ammonia formed upstream is reduced and urea can be quantified against a well‐defined nitrate baseline, ideally with ^15^N and/or ^13^C isotopic tracing to eliminate false positives.^[^ ^3^ ^]^

**Scheme 1:**
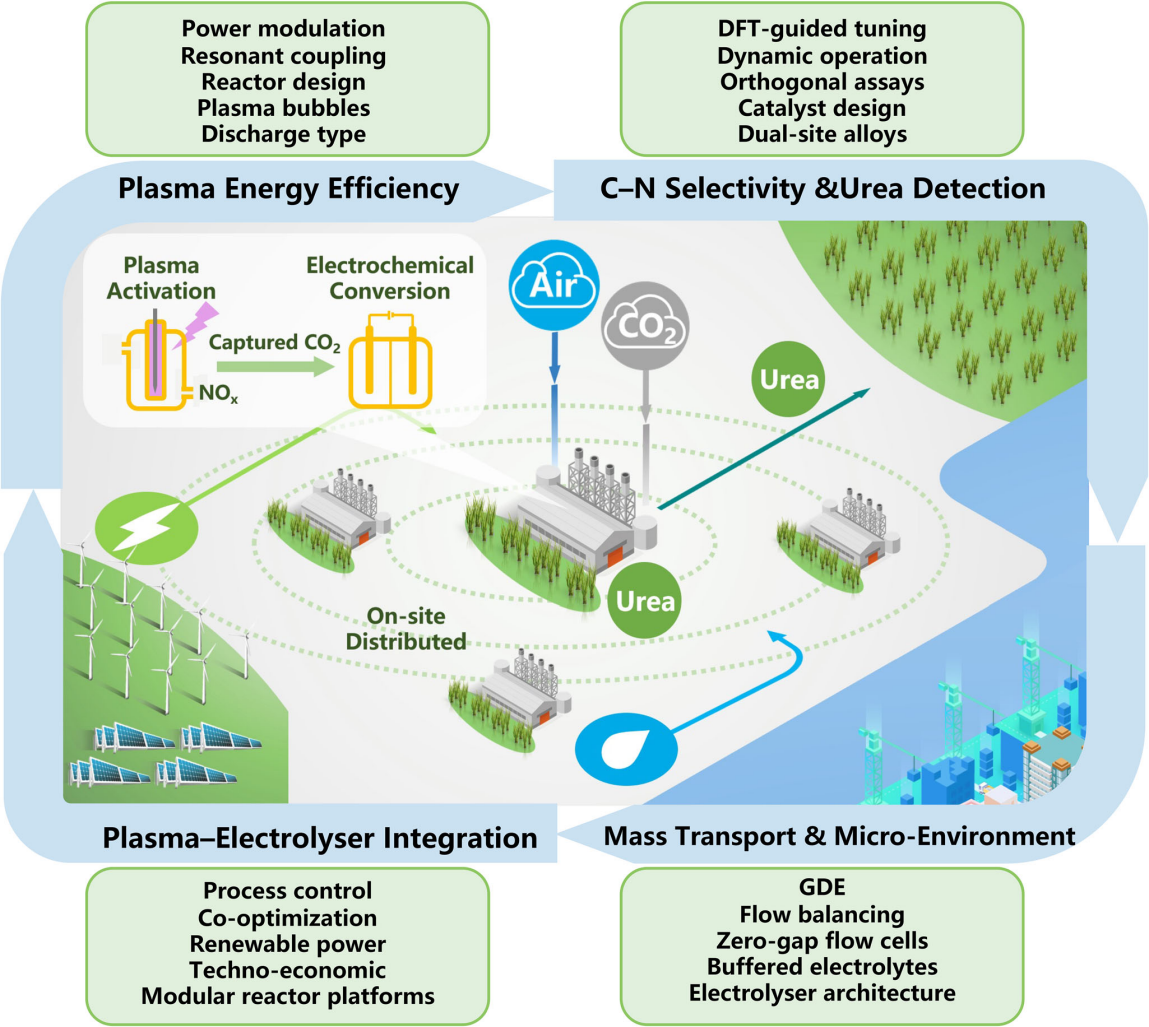
Key challenges and the roadmap for plasma‐enabled electrocatalysis in urea production.

Of course, the plasma‐electrolyzer relay presents its engineering challenges (**Scheme** **1**). The main engineering challenge is reducing the specific energy consumption of the plasma step. This requires effective reactor designs and power modulation strategies to minimize the energy input per mole of NO_x_ formed, while generating sufficient vibrationally and electronically excited molecules to maximize net nitrate production at scalable, industrially relevant rates. Kinetic modelling indicates that the theoretical minimum is ≈0.2 MJ mol^−^^1^.^[^ ^11^ ^]^ However, in practice, gas heating, quenching kinetics, backward reactions, and radiative losses result in most laboratory systems falling within the 1–3 MJ mol^−^^1^ range.^[^ ^12^ ^]^ Much of today's plasma research focuses on strategies that approach the asymptote, such as nanosecond pulsing to reduce electron‐ion recombination, resonant microwave coupling to promote selective vibrational pumping, micro‐bubble absorbers that rapidly quench NO_x_ in solution, and modular gliding‐arc arrays whose heat can be recovered downstream.^[^ ^8^, ^13^ ^]^ These innovations are more than just academic curiosity; they directly impact scalability, because energy efficiency, rather than capital cost, dominates the levelized cost of hybrid nitrogen chemistry once the reactors are mass‐produced.

The relay architecture presents a temporal coordination challenge: Nitrate production must match electrolyzer demand without relying on costly buffer tanks or oversized power electronics. Flow‐reactor modelling suggests that coupling a 2‐h nitrate inventory with feedback‐controlled plasma duty cycles can navigate typical renewable‐power ramps while adding only 3–5% to capital expenditure (capex).^[^ ^14^ ^]^ On the electrochemical side, the catalyst must co‐stabilize *CO and *NH_2_ long enough to facilitate the reaction. This requires 3D porous geometries, dual‐site alloys, and dynamic operating modes such as millisecond potential oscillations, which periodically enrich the surface in one intermediate before the other arrives.^[^ ^10^ ^]^

To determine the extent to which the field is already approaching commercial break‐even, we developed a first‐pass techno‐economic model that integrates experimentally demonstrated plasma power draws, electrolyzer metrics, and modular hardware costs into a single discounted‐cash‐flow framework. The exercise shows that as soon as i) plasma reactors fall below ≈ 1 MJ mol^−^^1^ NO_x_ (on a credible glide path to the 0.2 MJ mol^−^^1^ vibrational‐activation floor) and ii) flow‐cell cathodes deliver ≥ 100 mA cm^−^^2^ at ≥ 70% Faradaic efficiency (FE) (N‐selectivity ≥ 70%), the hybrid route can undercut the natural‐gas benchmark wherever unsubsidized RE averages ≤ US $35 MWh^−^^1^ (≈3.5 ¢ kWh^−^^1^), a tariff already standard during midday‐solar or night‐wind surpluses in Spain, Chile, California, and Western Australia.^[^ ^15^ ^]^ In other words, the focus is no longer on the sustainability of a staged plasma‐electrochemical pathway; rather, it is on the speed at which the community can develop the integrated, energy‐efficient designs necessary to advance it to the commercial stage.

After setting the stage in Section 1 by outlining both the opportunity and the remaining bottlenecks, the perspective proceeds downstream in a single, rising arc. Section 2 explores plasma micro‐kinetics, demonstrating how discharge geometry, nanosecond power modulation, plasma‐water interface, and high‐efficiency absorbers are already influencing NO_x_ energy yields in the direction of the sub‐megajoule regime. Section 3 follows the activated nitrogen as it enters the electrolyzer, first at the molecular level, demonstrating how dynamic biasing, vacancy/facet co‐engineering, and dual‐site catalysts orchestrate the critical *CO + *NH_2_ coupling. Subsequently, it applies these insights to the device level, transforming them into GDEs, zero‐gap MEAs, and plasma‐coupled flow cells resilient enough to withstand carbonate fouling. Section 4 integrates physics and chemistry into a comprehensive techno‐economic and life‐cycle framework, identifying the current and stretch “Plasma electrocatalysis (PE)‐Potential” scenarios in which cost and carbon parity emerge. Lastly, Section 5 distills the open scientific questions and engineering milestones into a forward‐looking research agenda, emphasizing the areas where the steepest learning curve gains will be achieved through coordinated progress in plasma science, electrocatalysis, and systems integration.

## 2 Plasma Discharge Engineering

In 1903, Kristian Birkeland and Samuel Eyde developed the plasma‐assisted nitrogen fixation process, known as the Birkeland–Eyde (B–E).^[^ ^6^ ^]^ They accomplished this by directing air through an electric arc within a magnetic field, thereby oxidizing nitrogen to generate nitrogen oxides, which were subsequently absorbed with water and converted into nitric acid. Nevertheless, the process was hindered by considerable energy inefficiencies. The majority of the energy input was ineffectively utilized for nitrogen fixation. The plasma operated at high temperatures (3000–10 000 K), resulting in a significant amount of energy being lost as thermal heat rather than being utilized to drive the desired chemical reactions. The excess heat also caused rapid decomposition of products, thereby degrading both the yield and efficiency of the process. This led to a nitrogen fixation efficiency of only 1–2 mol% and an energy consumption of 2.4–3.1 MJ mol^−^^1^, significantly higher than the more efficient Haber–Bosch process, which uses only 0.5–0.6 MJ mol^−^^1^.^[^ ^16^ ^]^ The B‐E process became uncompetitive due to high energy demand and low productivity, eventually replacing it by the Haber–Bosch method.

Nearly a century later, the advent of non‐thermal plasmas has changed this narrative. Non‐thermal plasmas operate under conditions that decouple the electron temperature from the gas temperature. In these plasmas, the electrons are much hotter (highly energized, 1–3 eV) than the gas, which remains at or near room temperature.^[^ ^17^ ^]^ This electron‐vibrational non‐equilibrium is crucial because it allows electrons to selectively excite nitrogen molecules into high vibrational states (*N_2 (v)_*) without heating the gas to high temperatures. These vibrationally excited nitrogen molecules are more reactive and can efficiently participate in the Zeldovich reactions (R_1_ to R_3_), which are not strongly temperature‐dependent.^[^ ^18^ ^]^

(1) $$N_{2}+e^{-}\to N_{2}+e^{-}\to \left(R_{1},\;vibrational\;excitation\right)$$

(2) $$N_{2\left(v\right)}+O\to \mathrm{NO}+N\to \left(R_{2},\;vibration\;activated\;Zeldovich\;step\right)$$

(3) $$N+O_{2\left(v\right)}\to \mathrm{NO}+O\to \left(R_{3},\;chain\;propagation\right)$$

In contrast, the high temperatures in thermal plasmas accelerate the back‐reactions (highly temperature dependent), leading to the destruction of NO back to N_2_ and O_2_. By maintaining a low gas temperature, non‐thermal plasmas kinetically suppress these reverse reactions and minimize vibrational‐to‐translational (V‐T) energy losses. As a result, non‐thermal plasmas are more likely to achieve significantly higher productivity and energy efficiency, as the forward reactions are more kinetically feasible, and undesirable back‐reactions are effectively minimized. Thus, this vibrational–translational non‐equilibrium allowed non‐thermal reactors to approach 1–3 MJ mol^−^^1^ today, with clear pathways (e.g., nanosecond pulsing, local‐field enhancement, rapid in situ quenching, microbubble absorbers) to push the energy toward the 0.2 MJ mol^−^^1^ vibrational activation floor.^[^ ^8^ ^]^

### 2.1 Plasma Assisted NO_x_ Generation

**Figure 1:**
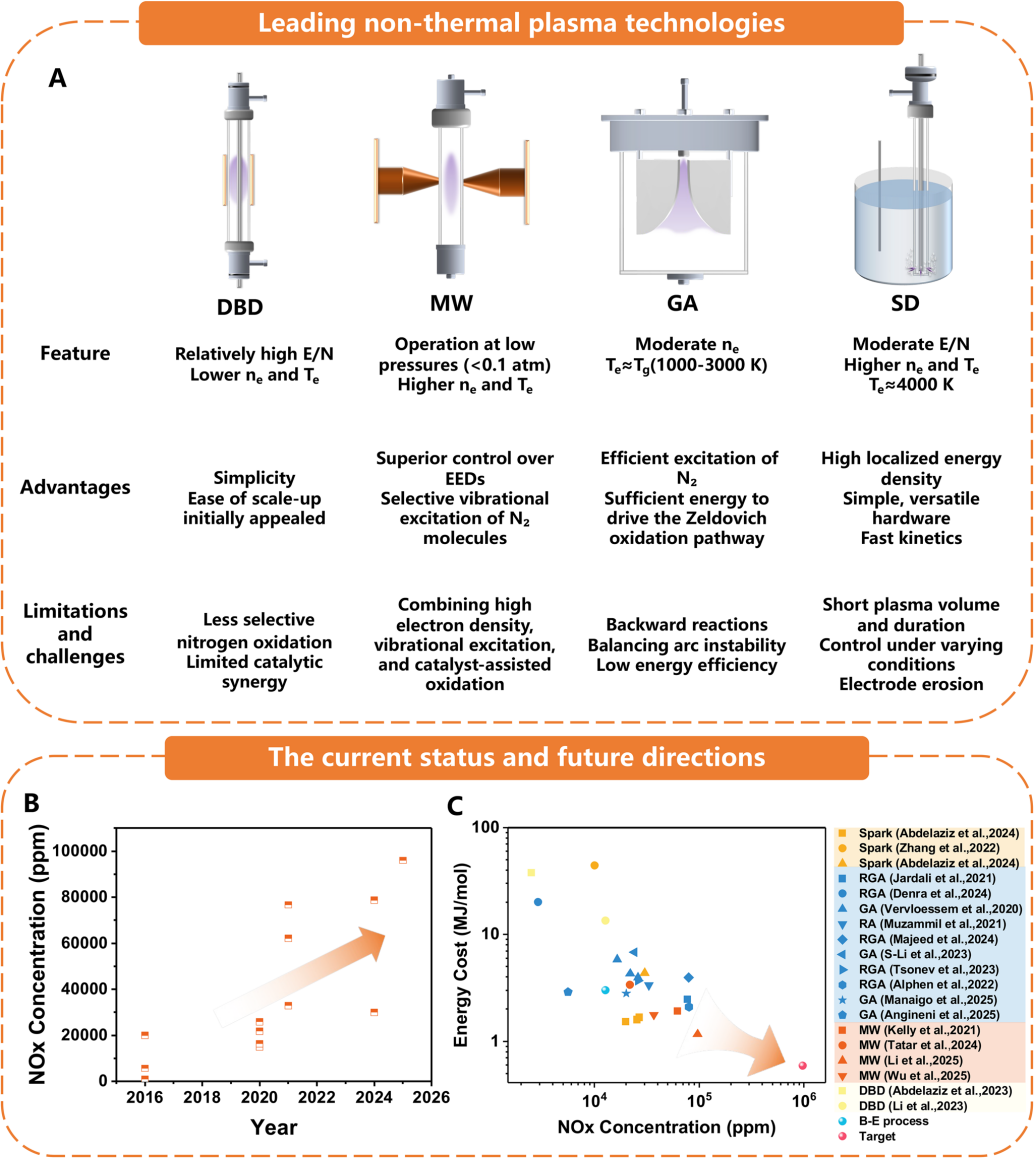
A) Schematic of various plasma types and their advantages, challenges, and opportunities. B) Reported NO_x_ concentrations over the last 5 to 10 years. C) Summary of plasma‐assisted nitrogen oxidation performance over the last five years (2020–2025). The source literature for the data points is organized in Supporting Information tables.^[^ ^12^, ^13^, ^23^, ^24^, ^26^, ^27^, ^28^, ^29^, ^30^, ^101^, ^102^, ^103^, ^104^, ^105^, ^106^, ^107^ ^]^ The target refers to the industrial Haber–Bosch method, which can utilize up to 0.6 MJ mol_N_^−^^1^ with a NO_x_ concentration of 972 711 ppm.

The four leading non‐thermal plasma technologies for oxidation‐based nitrogen fixation, DBD, microwave (MW), gliding arc (GA) plasmas, and spark discharge (SD) plasmas, each exhibit unique operational profiles that influence their energy efficiency, productivity, and scalability (**Figure** **1**).^[^ ^19^ ^]^ In the following sections, we will briefly examine these characteristics to determine which approach offers the most promising pathway for seamless integration with electrocatalytic C─N bond formation.

DBD reactors operate at relatively high reduced electric fields but have lower electron densities and temperatures than microwave and gliding arc plasmas.^[^ ^20^ ^]^ While their simplicity and ease of scale‐up initially appealed, their low electron temperatures result in less selective nitrogen oxidation, resulting in significant ozone production and correspondingly lower NO yields.^[^ ^8^ ^]^ Despite efforts to incorporate catalysts into DBD systems to improve fixation efficiency, catalytic synergy is still limited, frequently introducing hidden energy costs and environmental complications in catalyst synthesis.^[^ ^21^ ^]^ These inherent limitations have gradually shifted research attention away from DBD and toward plasma sources that can better harness electron energy for more selective nitrogen oxidation pathways.^[^ ^13^, ^22^ ^]^ Thus, DBD might still play a role in systems where simplicity and scalability are prioritized; however, its limitations in selectivity suggest that it would need integration with a more efficient electrocatalytic process for higher productivity and energy savings.

MW plasmas, such as electron cyclotron resonance and surface wave discharges, offer superior control over electron energy distributions, resulting in higher electron temperatures and densities required for selective vibrational excitation of nitrogen molecules. MW plasmas typically operate at low pressures (<0.1 atm) to maximize electron mean free paths and energy efficiency.^[^ ^13^ ^]^ However, the additional energy costs associated with vacuum infrastructure can reduce net efficiency gains. Wu et al. utilized a tailored tungsten rod array to enhance the local electric field to 2.7 × 10^7^ V m^−^^1^, 385 times higher than conventional configurations. This resulted in a significant reduction in energy consumption (down to 1.78 MJ mol^−^^1^) and an increase in NO and NO_2_ (NO_x_) yields (3.68%).^[^ ^23^ ^]^ Li et al. enhanced MW plasma systems with WO_3_/HZSM‐5 catalysts, achieving up to 9.6% NO_2_ concentrations at practical gas throughputs (15–30 L min^−^^1^) with a low energy cost of 1.17 MJ mol^−^^1^.^[^ ^24^ ^]^ These studies found that combining high electron density, vibrational excitation, and catalyst‐assisted oxidation resulted in exceptional NO_x_ production metrics, indicating a promising path for scalable and efficient plasma‐based nitrogen fixation. To this end, MW plasmas show great promise for efficient nitrogen fixation, and their integration with electrocatalysis for urea production could offer a scalable solution. The optimization of MW plasma systems with catalysts could provide the selectivity and energy efficiency necessary for the hybrid process.

GA plasmas, known as “warm plasmas,” are an intermediate regime between cold (DBD) and fully thermal plasma systems. GA plasmas typically have moderate electron densities and electron temperatures close to the gas temperature (1000–3000 K).^[^ ^25^ ^]^ This operating regime efficiently excites N_2_ molecules while maintaining enough energy to drive the Zeldovich oxidation pathways effectively. Despite these favorable conditions, GA plasmas have distinct limitations that prevent them from operating at peak efficiency. For example, elevated gas temperatures downstream of the plasma zone can cause undesirable backward reactions, such as those associated with thermal plasma.^[^ ^26^ ^]^ Second, balancing arc stability and energy efficiency is a persistent challenge. Manaigo et al. found that adding external resistors (1–19 kΩ) to GA systems stabilized the plasma discharge by suppressing rapid arc fluctuations, reducing current instability from 28% to 3.7%. Despite the improved plasma stability, Joule heating from resistors resulted in an undesirable increase in energy consumption from 2.82 to 7.9 MJ mol^−^^1^, a 180% increase. Replacing resistors with a 100 mH inductor reduced high‐frequency fluctuations without compromising energy efficiency. Energy costs remained close to the original 2.82 MJ mol^−^^1^, with comparable NO_x_ yields of ≈2%.^[^ ^27^ ^]^ Nonetheless, more research is needed to fully understand the impact of such electrical modifications on plasma chemistry and reaction kinetics. Angineni et al. addressed the productivity and efficiency limitations of GA plasmas by operating at higher pressures. By increasing the pressure to 2 bar, gas molecules collide more frequently, resulting in an 84% selectivity for NO_2_ and yields of 10.2 and 8.83 g h^−^^1^, respectively, at an energy cost of 2.9 MJ mol^−^^1^.^[^ ^28^ ^]^ Therefore, GA plasmas, when optimized to minimize thermal losses and mitigate reverse reactions, can complement electrocatalytic processes, particularly when higher throughput is required. However, their high energy consumption needs to be addressed for scalability.

The rotating gliding arc (RGA) is a 3D evolution of GA plasma, which overcomes the GA limitations by expanding the reaction zone and achieving a more uniform distribution of reactive species. Introducing a magnetic field further enhances arc rotation, increases gas‐arc interaction time, and improves energy utilization. The combined influence of aerodynamic and Lorentz forces maintains the arc's stable rotation. Flow‐ and magnetically driven RGA systems have become a research focus due to their superior performance. Jardali et al. designed a rotating sliding arc discharge device that can operate in either a rotating or stable‐arc mode. In the stable‐arc mode, the NO_x_ volume fraction reaches 5.5 %, 1.7 times higher than in the rotating‐arc mode, with an energy consumption of 2.5 MJ mol^−^^1^. Simulations indicate that the higher NO_x_ concentration results from the thermal plasma region occupying most of the reactor volume, allowing gas molecules to remain under plasma treatment for a longer residence time. By contrast, in rotating‐arc mode, NO_x_ forms mainly via vibration‐promoted Zeldovich reactions, and the rapid cooling of gas outside the arc suppresses the relaxation of vibrationally excited species.^[^ ^26^ ^]^ Later, Alphen et al. improved the device by adding a quench nozzle, raising the NO_x_ volume fraction to 5.9 % while reducing energy consumption to 2.1 MJ mol^−^^1^. The quench nozzle rapidly cools the gas after it leaves the plasma region, preventing reverse reactions that could occur at high plasma temperatures.^[^ ^12c^ ^]^

Spark discharges feature extremely high local electron densities (10^16^–10^19^ cm^−^^3^) and “warm” plasmas with gas (rotational) temperatures of roughly 1400–2600 K, vibrational temperatures ≈3700–4300 K; they require electric fields exceeding the breakdown threshold in air (≈30 kV cm^−^^1^) and often operate between 10^6^ and 10^7^ V m^−^^1^. Their high localized energy density and simple hardware make them ideal for rapid nanoparticle synthesis and plasma chemistry, offering fast heating and cooling kinetics, as well as versatile material processing. However, spark discharges suffer from low overall energy efficiency and limited product selectivity, rapid electrode erosion under repeated pulsing, sensitivity to pressure, gas composition, and geometry, which complicates stable operation, as well as inherently short plasma volumes and lifetimes that challenge continuous, large‐scale applications. Abdelaziz et al. systematically investigated spark discharge to identify factors governing efficiency and propose strategies for improved energy use. Applying high‐frequency bipolar voltage enhances NO_x_ yield and efficiency by utilizing residual species. Active cooling exhibited minimal chemical losses, with electrode heating, particularly above 20 kHz, being the primary source of energy loss, especially in the spark anchor state. However, higher frequencies offset this by slowing the rise in energy cost with increasing energy density. Expanding the plasma zone by increasing the electrode gap improved energy utilization and adding a floating electrode enabled higher NO_x_ production (1.8–3.0%) at lower energy costs (1.9–4.4  MJ mol^−^^1^). Optimizing reactor design could further enhance performance. These results provide a strong foundation for advancing plasma reactors in efficient chemical synthesis.^[^ ^29^ ^]^ Zhang et al. developed a nanosecond pulsed spark discharge system with a plate‐to‐plate configuration for sustainable nitrogen fixation under ambient conditions. NO_x_ concentrations of 960–10 900 ppm and energy efficiencies of 12.4–34.2 MJ mol^−^^1^ were achieved across airflow rates of 40–340 mL min^−^^1^. Optical emission spectroscopy and chemical kinetics modeling revealed that NO_x_ formation pathways are highly sensitive to plasma conditions and species residence time. Over 50% of NO was generated via chain reactions involving O and N radicals with vibrationally excited N_2_ and O_2_, while NO_2_ primarily formed through subsequent oxidation (NO + O → NO_2_). The detection of O and N spectral lines in the post‐discharge phase further confirmed the dominant role of free‐radical chain reactions.^[^ ^30^ ^]^ In recent years, spark discharge has been increasingly applied in gas–liquid discharge‐based nitrogen fixation, which will be discussed in the following section.

### 2.2 Liquid‐Phase NO_x_ Generation and Absorption

**Figure 2:**
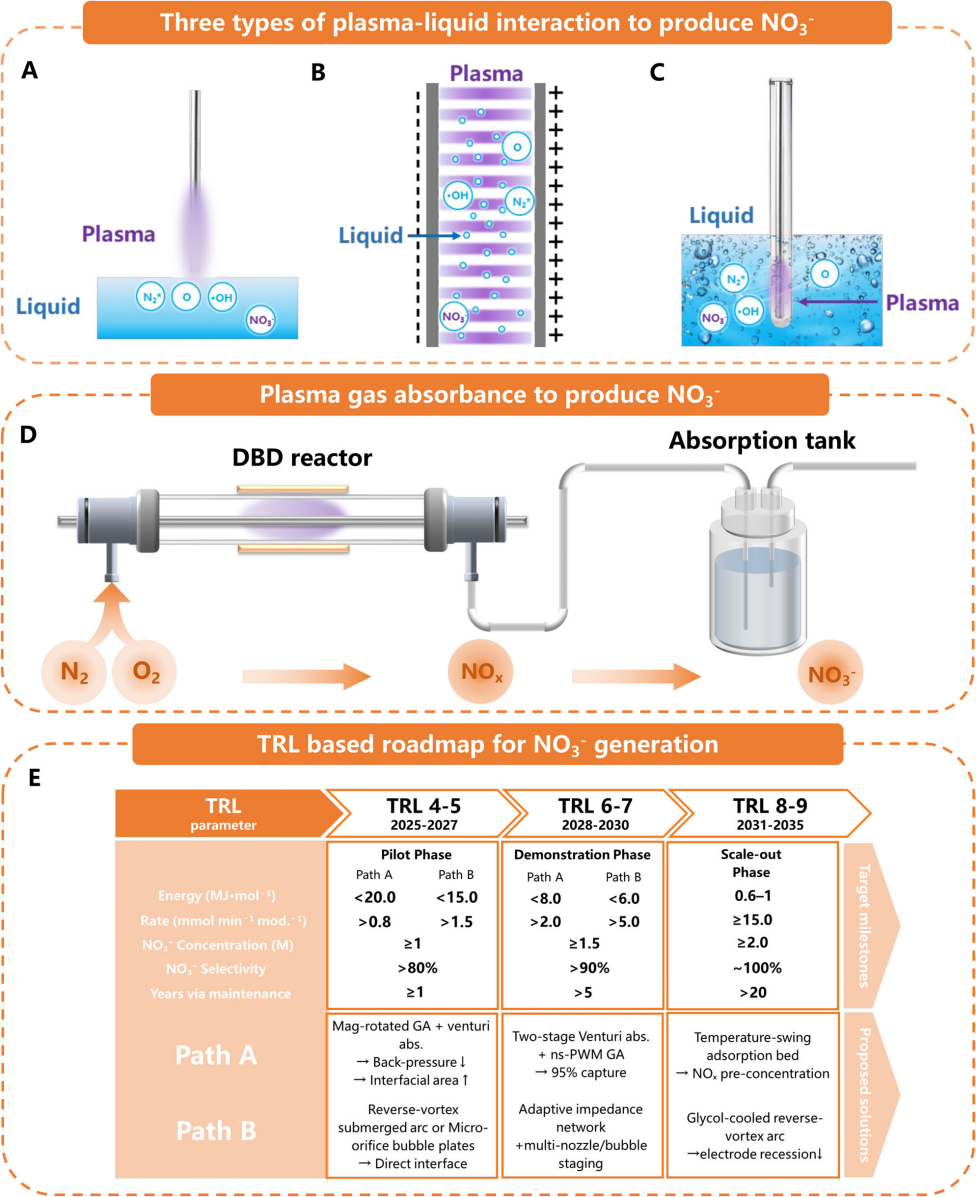
The schematics of liquid‐phase NO_x_ generation and absorption. A) Plasma‐on‐liquid type. B) Liquid‐in‐plasma type. C) Plasma‐in‐liquid type. D) Indirect methods involving gas‐phase NO_x_ generation followed by absorption into water. E) Roadmap for advancing the TRL of plasma‐assisted nitrate generation. Path A couples a small gas‐phase reactor to a venturi micro‐bubble absorber, while Path B forms nitrate directly in water with a submerged or surface discharge. Module size (plasma volume ≈80–120 cm^3^, power ≤ 2 kW) is fixed; higher TRLs are reached by boosting energy efficiency, selectivity, and durability, so plant capacity is expanded simply by stacking identical slabs.

For effective integration with electrocatalytic urea synthesis systems, plasma‐generated nitrogen oxides must be efficiently captured into aqueous solutions, typically in the form of nitrate and nitrite ions. Currently, two primary approaches are shown schematically in **Figure** **2**: 1) indirect methods involving gas‐phase NO_x_ generation followed by absorption into water and 2) direct plasma discharge at the gas‐liquid interface. Each approach presents different challenges and opportunities.

In the indirect approach, incomplete absorption limits efficiency, resulting in significant NO_x_ loss through off‐gassing and energy inefficiencies. To address these limitations, Lan et al. developed a gas‐liquid two‐phase flow system that uses a Venturi tube to generate microbubbles, resulting in significantly improved gas–liquid mass transfer. This design significantly improved NO_x_ absorption efficiency, resulting in a 33.7% reduction in energy consumption (from 50.65 to 17.48 MJ mol^−^^1^) and a 52.8% increase in nitrate yield compared to traditional bubble absorption methods.^[^ ^7a^ ^]^ However, the additional energy required to operate the Venturi tube and manage the two‐phase flow emphasizes the ongoing need for further optimization of gas‐liquid interaction techniques.

The alternative direct plasma discharge at the gas‐liquid interface has inherent challenges because water molecules quench vibrationally excited nitrogen species. V‐T energy transfer in water inhibits the desired vibrational‐to‐vibrational (V‐V) energy exchanges required for efficient nitrogen fixation. Nonetheless, recent innovations have helped to advance this approach. Zhou et al. produced an underwater multi‐bubble plasma reactor capable of operating in multiple discharge modes (DBD, spark, and hybrid), demonstrating that adjusting electrical parameters and using catalytic materials significantly increased nitrate yields. This reactor achieved high liquid‐phase nitrate production efficiency at a competitive energy cost of 9.7 MJ mol^−^^1^, considerably improving over previous gas‐liquid interface plasma systems.^[^ ^8^ ^]^ Building on this, Zhou et al. improved the design by incorporating a nanosecond pulsed liquid surface discharge reactor with coplanar plate electrodes and conical insulating structures, thereby increasing the discharge area and stability. Systematic adjustments to electrical and solution parameters resulted in nitrate concentrations of up to 1.43 mM at 14.74 MJ mol^−^^1^, or ≈30% less energy than comparable technologies.^[^ ^31^ ^]^

Zhang et al. demonstrated another highly promising system, a submerged transferred‐arc gliding arc plasma reactor with reverse vortex flows. The researchers found stable underwater plasma formation above a current threshold of 0.6 A, resulting in high throughput with NO_3_^−^ concentrations reaching 2.4% and nitrogen fixation energy costs as low as 2.21 MJ mol^−^^1^. This was the lowest energy cost ever achieved for atmospheric‐pressure gliding arc plasmas, demonstrating the potential of optimized gas‐liquid interface reactors to meet critical productivity and efficiency benchmarks.^[^ ^32^ ^]^

### 2.3 Scale‑Up Roadmap and Technology Readiness Level (TRL) Milestones for Plasma Reactors

Non‑thermal plasma modules for hybrid nitrate‑to‑urea synthesis advance along two complementary engineering routes. Path A confines the discharge to the gas phase, implemented as a magnetically steered rotating‑gliding arc, a nanosecond‑reignited spark train, or a low‑pressure microwave cavity, and couples it to an energy‑recovered venturi micro‑bubble absorber.^[^ ^7^, ^13^, ^30^ ^]^ Path B ignites the plasma within, or directly above, the aqueous phase, for example through a reverse‑vortex submerged transferred arc or micro‑orifice bubble columns, enabling in situ nitrate formation and immediate quenching.^[^ ^32^, ^33^ ^]^ Industrial capacity is unlocked by numbering‑up these optimised cassettes rather than by enlarging any single reactor. Accordingly, TRL progression is judged by gains in specific energy, nitrate selectivity, and service life while cassette footprint (≈80–120 cm^3^ plasma volume) and power draw (0.2–2 kW across the TRL ladder) remain within a compact envelope. Performance improvements therefore, derive solely from tighter control of electron‑energy distributions, enhanced gas–liquid mass transfer, and erosion‑resistant materials, not from geometric scale (Figure 2E).

#### 2.3.1 TRL 4‑5: Pilot‑Prototype Validation

Bench‐scale reactors are repackaged as compact slabs, drawing 0.2–0.5 kW. At this stage, each cassette must deliver 0.8–2 mmol NO_x_ min^−^^1^, which, after auxiliary loads for micro‑bubble sparging or liquid recirculation, translates to a cradle‑to‑gate energy figure of 15–20 MJ mol^−^^1^ referenced to nitrate. In Path A, this figure is dominated by absorber hydraulics: a tri‑nozzle, magnetically rotated gliding‑arc head run under nanosecond gating may achieve 2–3 MJ mol^−^^1^ at the plasma stage yet coupling it to a first‑generation venturi micro‑bubble column presently adds ≈17 MJ mol^−^^1^. The engineering emphasis is therefore on reducing liquid back‑pressure (target <20 kPa) and improving gas–liquid interfacial area so that capture surpasses eighty percent without inflating auxiliary load. Path B circumvents an external absorber by interfacing the discharge directly with the electrolyte through reverse‑vortex submerged arcs or micro‑orifice bubble plates. Here, the plasma section already reaches 2–3 MJ mol^−^^1^ NO_x_, but the overall energy per mole of nitrate remains 9–15 MJ mol^−^^1^ because only 30–50 % of the nitrogen ends as nitrate and vigorous circulation is required to dissipate heat. Across both pathways, the outlet liquor must nevertheless reach ≥1 M nitrate so that the downstream urea electrolyzer can operate without additional concentration steps, establishing the baseline against which subsequent TRL gains will be measured.

#### 2.3.2 TRL 6‑7: Demonstration Under Industrial Duty Cycles

Multiple‑cassette clusters, typically ten to fifteen modules, each drawing 0.2–0.5 kW, for an aggregate plasma load of ≈ 2–7 kW, are coupled to a pilot nitrate‑to‑urea electrolyzer and operated continuously for 90 days while tracking photovoltaic power ramps. To clear this milestone, the cluster‑averaged, cradle‑to‑gate specific energy must fall below 6 MJ mol^−^^1^ NO_3_^−^, overall nitrate selectivity must exceed 90 %, and the product stream must stabilize at ≥2 M nitrate with <3 % drift during load‑following. In Path A, these thresholds are met by a two‑stage, counter‑current Venturi absorber that completes in‑flight NO→NO_2_ oxidation and lifts gas‑to‑liquid capture to 95 % while keeping hydraulic losses <15 kPa; a nanosecond pulse‑width‑modulated gliding arc synchronized to magnetic rotation maintains a high electron temperature yet curbs bulk gas heating. Path B integrates an adaptive impedance network that corrects ±10 % conductivity swings within 50 ms, suppresses arc‑root hopping, and restrains electrode recession to <0.3 µm h^−^^1^, enabling > 10 000 h of uninterrupted operation. Although superficial gas velocity remains 2.5 m s^−^^1^, internal multi‑nozzle or multi‑bubble staging doubles residence time without enlarging the cassette footprint, keeping the cluster within standard rack dimensions.

#### 2.3.3 TRL 8‑9: Bankable Module Qualification

The industrial‑grade cassette retains the reactor size and plasma volume unchanged but, through internal optimization, must now furnish ≥15 mmol NO_3_^−^ min^−^^1^ (i.e., 0.9 mol h^−^^1^) at a cradle‑to‑gate specific energy of 0.6–1 MJ mol^−^^1^, realize ≈100% gas‑to‑liquid capture, and export liquor at ≥2 M nitrate without auxiliary concentration. Each cassette thereby becomes a self‐contained, high‐performance “Lego block” whose simple replication, rather than redesign, scales plant throughput while unit cost falls with volume manufacturing. Path A reaches these metrics by magnetically pinning the rotating‑gliding arc inside a coaxial stainless cavity and driving it with a purpose‑built, nanosecond pulser whose voltage, frequency, and duty are factory‑set to the cassette impedance. Exhaust NO_x_ is pre‑concentrated on a BaO temperature‑swing bed, trimming vacuum‑pump work to <0.05 MJ mol^−^^1^, after which a two‑stage Venturi (ΔP ≈ 15 kPa) secures near‑quantitative capture. Path B mounts a reverse‑vortex submerged arc on a copper‑alloy baseplate cooled by glycol plates so that wall temperature remains <1000 K, holding electrode recession below 0.2 µm h^−^^1^ and extending unattended service intervals beyond 40 000 h. An adaptive impedance network sustains a power factor of > 0.93 through ±30 % renewable‑power swings. A compact, plug‑in power‑supply cartridge fixed to each reactor body delivers the optimized waveform, enabling mass production of an integrated plasma‑plus‑driver unit that is safe to stack on a common DC bus. Slide‐out electrode cassettes and quick‐disconnect coolant manifolds enable hot‐swap maintenance, supporting a 20‐year design life with scheduled annual servicing and minimal downtime. Finally, cloud‑hosted AI schedulers forecast renewable generation and pre‑programmed cassette duty cycles, maximizing utilization during low‑cost electricity windows while preserving component longevity.

## 3 Electrocatalytic NO_3_^−^/CO_2_ Coupling for C─N Bond Formation

**Figure 3:**
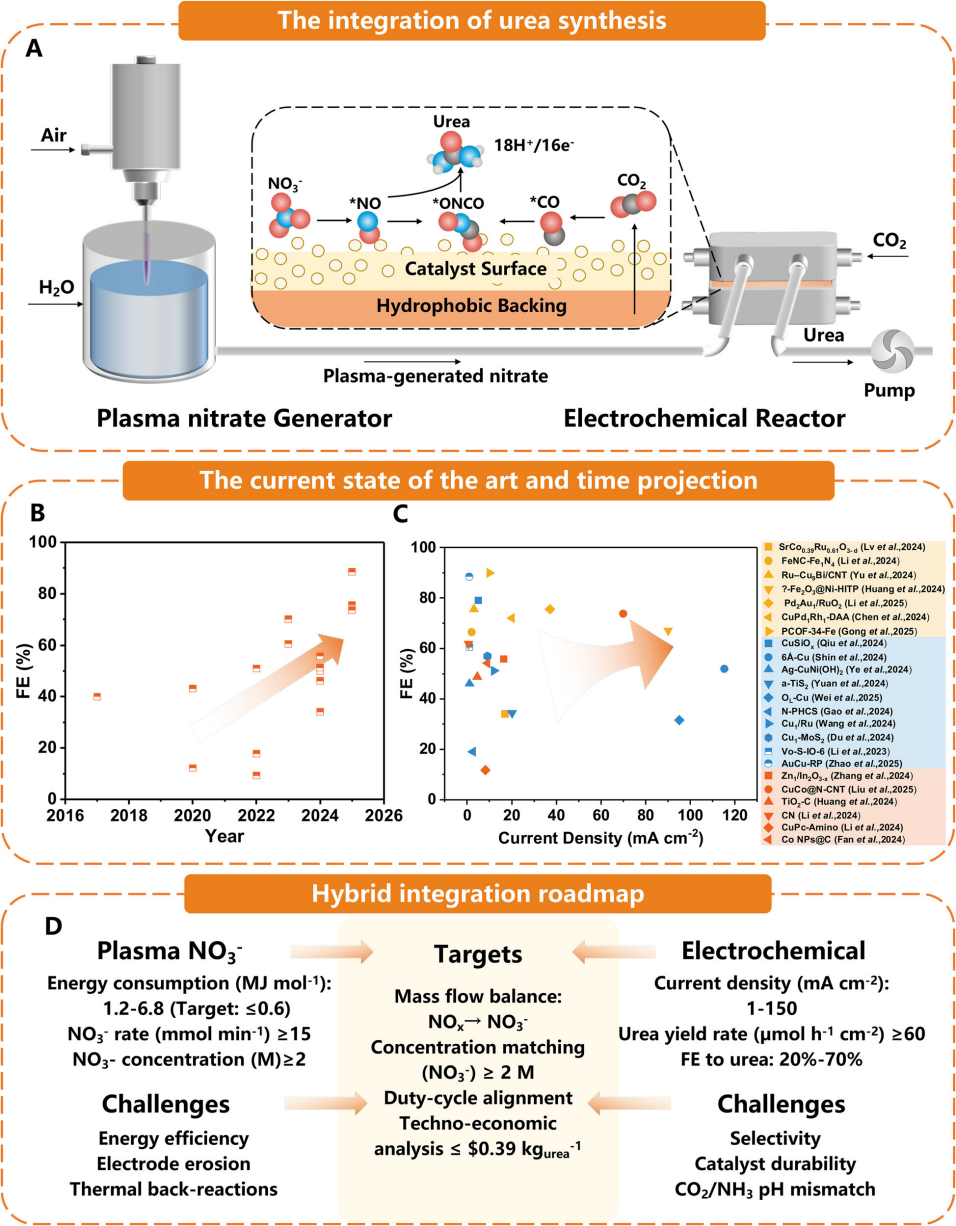
A) The schematic of system integration. B) The time projection of FEs for electrochemical synthesis of urea. C) The current state of the art of electrocatalytic NO_3_^−^/CO_2_ coupling for urea formation.^[^ ^34^, ^35^, ^38^, ^39^, ^41^, ^43^, ^48^, ^57^, ^108^, ^109^, ^110^, ^111^, ^112^, ^113^, ^114^, ^115^, ^116^, ^117^, ^118^, ^119^, ^120^, ^121^, ^122^ ^]^ The legend is arranged in descending order of durability time. Yellow indicates 100–150 h, blue indicates 50–100 h, and orange indicates 1–50 h. D) A hybrid integration roadmap, challenges, and targets.

These recent advances highlight clear opportunities and ongoing challenges for non‐thermal plasma nitrogen fixation. While non‐thermal plasma systems provide stable, high‐throughput operation at reasonable energy costs, further electrical efficiency and reactor design improvements are required. Liquid‐phase NO_x_ generation and absorption strategies should optimize mass transfer efficiency and reduce auxiliary energy demands to ensure economic viability. Advanced reactor engineering, power modulation techniques, and catalytic enhancement will be critical in developing these plasma‐based nitrogen fixation methods into economically viable modules that can be integrated into a hybrid plasma‐electrocatalytic system for decentralized, sustainable urea production (**Figure** **3**).

The electrochemical coupling of plasma‐generated nitrate (NO_3_^−^) with CO_2_ (in an electrolyte‐saturated solution) is fundamental to C─N bond formation in the hybrid strategy. While bypassing the thermodynamically challenging direct N_2_ activation step, this approach presents its own complex chemical challenges. The electrocatalytic reaction involves a 16‐electron and 18‐proton proton–electron coupled transfer sequence (R_4_), underscoring the intricate complexity and the need for careful catalyst modulation.

(4) $$2N{O_{3}}^{-}+CO_{2}+18H^{+}+16e^{-}\to NH_{2}\mathrm{CON}H_{2}+7H_{2}O$$

The fate of every electron and proton supplied in the 16e^−^/18H⁺ cascade is determined by a number of surface‐bound intermediates. Operando spectroscopy and density‐functional theory now converge on four motifs: *NO, *NH, *OCNO (or its protonated counterpart *NHCOO), and the subsequent *NH–CO adduct, as critical points of control along the nitrate/CO_2_ pathway to urea. *OCNO, formed from the combination of *CO and *NO/*NO_2_‐derived intermediates, resides in a small free‐energy well and is consistently identified as the most thermodynamically favored C─N coupling product on Cu, PdAu, NiCo_2_O_4_, and indium‐oxide surfaces. However, the ensuing hydrogenations that transform *OCNO into *NH–CO and ultimately into urea still encounter barriers of at least 0.4 eV on most catalysts, whereas the off‐pathway protonation of *NO to NH_3_ or the competitive protonation of *CO to CO/formate remains feasible.

An effective cathode material must generate a high local coverage of *CO and *NO‐derived species, confine them long enough to form *OCNO, and modulate the electronic structure to favor *OCNO hydrogenation over parasitic Hydrogen evolution reaction (HER) and NH_3_ channels, resulting in selective intermediate choreography. Dual‐site motifs, such as Cu‐Zn, Cu‐In, Pd‐Au alloys, or spinel Ni‐Co oxides, provide complementary binding sites that meet these requirements. Additionally, defect‐rich carbon scaffolds or oxygen‐vacancy‐patched oxides can enhance adsorption strength without causing surface poisoning.

Electrolyte engineering, including buffer strength, cation identity, and the balance of hydrophobic and hydrophilic properties, along with local pH regulation and gas‐diffusion architectures, are critical factors that determine the residence time and speciation of solution‐phase NO_3_^−^/NO_2_^−^ and dissolved CO_2_. Suppressing local acidification retards *NO protonation to NH_3_, while mildly alkaline boundary layers stabilize *NO_x_‐derived fragments and enhance *OCNO formation. Hydrophobic GDEs effectively reduce flooding, sustain CO_2_ availability, and shift the competitive advantage away from HER.

### 3.1 Electrocatalyst Design

By removing us from the thermodynamic tyranny of N≡N cleavage, the plasma stage assigns the cathode a more complex task: coordinating two C─N couplings that weave CO_2_‐ and NO_x_‐derived fragments into urea. The first C─N bond can form via two classic routes. The Eley–Rideal mechanism involves a surface‐bound nitrogenous moiety (N_1_═*NO_2_, *NH_2_, etc.) attacking dissolved or gas‐phase CO_2_ through a single nucleophilic strike. The Langmuir–Hinshelwood mechanism involves pre‐adsorption of both partners, the carbon fragment (*CO or *COOH) and the nitrogen fragment (N_1_), followed by lateral diffusion to align them for coupling. Regardless of the path taken, both channels converge at the same surface bottleneck: the formation of the *OCNO adduct (or its protonated analogue, *NHCOO), which has been identified by operando spectroscopy and constant‐potential density functional theory (DFT) as the deepest free‐energy well on almost every catalyst studied to date. We will now discuss the fundamental principles of catalyst design and, subsequently, the strategies for controlling, timing, and stabilizing that chemistry under operating conditions.

#### 3.1.1 Fundamental Catalyst Design

**Figure 4:**
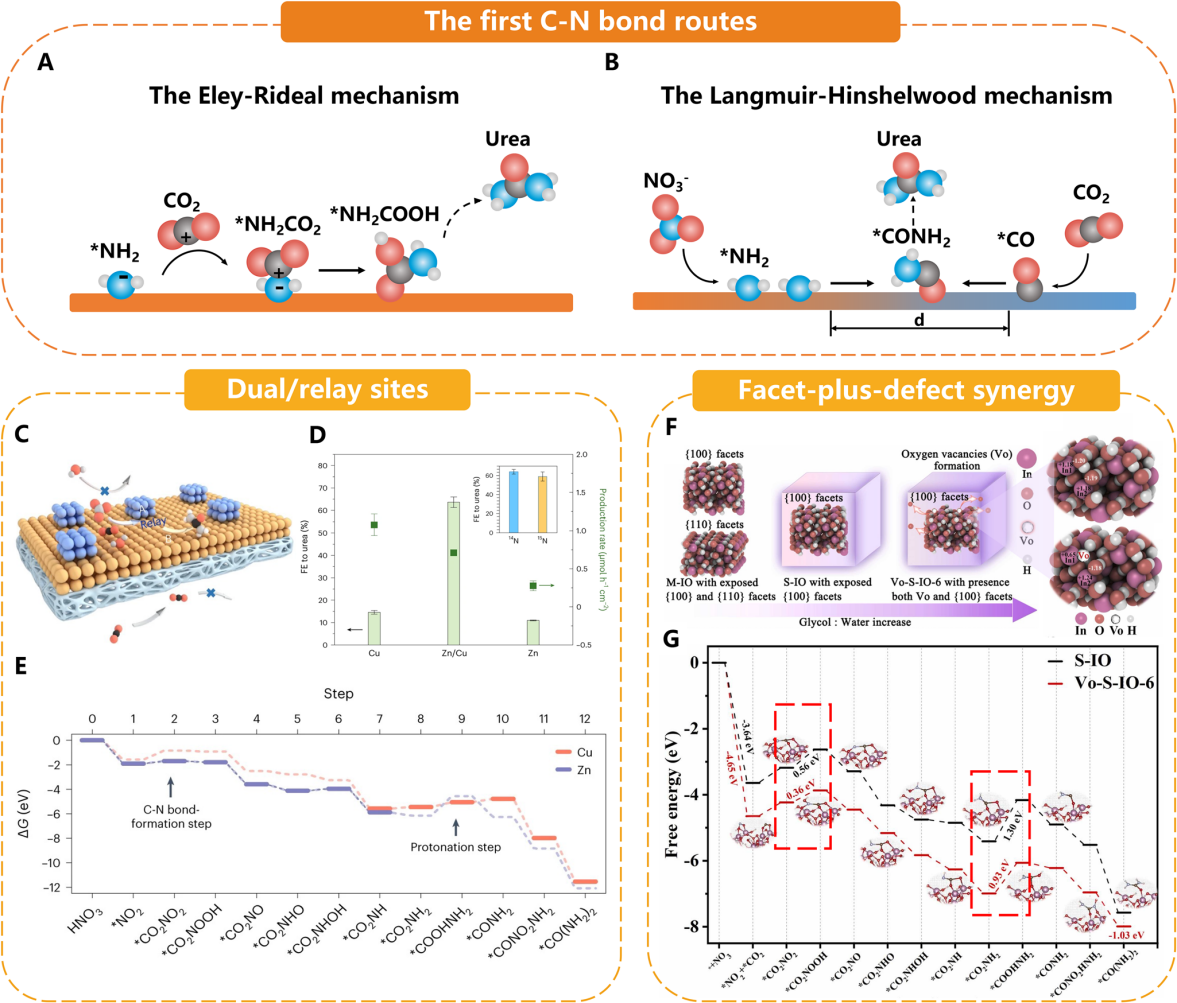
A) The Eley–Rideal mechanism and B) the Langmuir–Hinshelwood mechanism of the first C─N bond. C) The mechanism of a Zn/Cu hybrid catalyst, where one component (blue) lowers the reaction energy of the C─N bond‐formation step, and a second component (orange) reduces the reaction energy of the protonation step needed to produce urea. Red, dark, grey, and white denote O, C, N, and H. Reproduced with permission.^[^ ^5^ ^]^ Copyright 2023, Springer Nature. D) FEs to urea on the Zn/Cu hybrid catalyst and single‐component counterparts. Reproduced with permission.^[^ ^5^ ^]^ Copyright 2023, Springer Nature. E) DFT calculations of the free‐energy diagram for the synthesis of urea from NO_3_^−^ and CO_2_. Reproduced with permission.^[^ ^5^ ^]^ Copyright 2023, Springer Nature. F) Schematic diagram for the preparation of M‐IO, S‐IO, and Vo‐S‐IO‐6, and Bader charge analysis of S‐IO and Vo‐S‐IO‐6. Reproduced with permission.^[^ ^35^ ^]^ Copyright 2023, Elsevier. G) Free energy plots of S‐IO and Vo‐S‐IO‐6 for the electrosynthesis of urea at 0 V versus RHE. The red box labels the two protonation processes. Reproduced with permission.^[^ ^35^ ^]^ Copyright 2023, Elsevier.

Fundamental catalyst design comprises strategies that tailor the catalyst's surface environment to overcome the deep *OCNO energy well and drive the first C─N coupling step. This section discusses three core approaches, electrocatalyst design overview, dual/relay site architectures, and facet–defect synergy. It outlines the guiding principles and quantitative targets that unify these design rules, as demonstrated in **Figure** **4**.

##### Dual/Relay Sites: Breaking Linear‐Scaling Limits on the First C─N Bond

On a single metal surface, *CO and *NH_2_ bind through similar metal‐d orbital interactions. Consequently, the catalyst is pinned to a “Goldilocks” line that caps urea selectivity, as the strengthening of one almost inevitably over‐stabilizes the other. Bimetallic relay architectures avoid the linear‐scaling trap by assigning the two intermediates to electronically distinct neighbors. The Zn/Cu GDE reported by Luo et al. epitomizes the concept. Zn lowers the energy of forming *CO_2_NO_2_ (the first C─N adduct) by ≈0.4 eV relative to Cu, while Cu's higher d‐band center reduces the barrier for protonating *CO_2_NH_2_ to *COOHNH_2_ by more than 1 eV. By allowing each metal to specialize, the hybrid achieves 75% FE at 100 mA cm^−^^2^, three times the selectivity of the best single‐component analogues at wastewater‐level nitrate concentrations.^[^ ^5^ ^]^ The Pd_2_Au_1_/RuO_2_ heterostructure takes a different approach to the strategy. Alloying Pd with Au reduces the work‐function gap to RuO_2_ from 0.44 to 0.05 eV, removing excess interfacial charge and loosening. Spill‐over CO migrates tens of nanometers to Ru‐bound *NH_2_, increasing urea selectivity to 75.6% and lowering energy costs to 18.9 kWh kg_urea_^−^^1^.^[^ ^34^ ^]^

The following examples illustrate design logic for relay sites: i) Select metals (or oxide/metal pairs) with complementary affinities to anchor the C‐fragment and the N‐fragment; ii) keep their spacing within the diffusion length of *CO (<5 nm) but far enough apart to avoid µ_2_‐bridge CO that poisons the second C─N coupling; and iii) create an electronic gradient through alloying or oxide support to encourage directional migration rather than random surface hopping. In practice, relay catalysts consistently lower the first‐bond formation free energy below 0.6 eV and the subsequent protonation barrier below 0.5 eV, targets that single‐site oxides rarely achieve. Anchoring the catalyst section with this principle paves the way for the following levers, facet/defect tuning and real‐space spacing control, accelerating *OCNO processing toward urea.

##### Facet‐Plus‐Defect Synergy: Lowering the OCNO → NH–CO Protonation Barrier

Although uniform crystal facets provide ordered ensembles of metal–oxygen linkages that can cradle the *OCNO adduct in a well‐defined geometry, they rarely provide the electron flexibility necessary to push the adduct over its ≥0.4 eV protonation hurdle. Injecting a calibrated density of oxygen vacancies into such facets solves the problem by withdrawing electron density from neighboring cations, weakening the M–O bond to the *CO_2_NH_2_ group, and polarizing the O‐terminus to accept a proton more readily. Li et al.’s indium oxyhydroxide nano‐cubes demonstrate the effect spectacularly. Restricting growth to the {100} plane aligns In‐O‐x‐O‐In rows that template direct *CO_2_‐*NO_2_ coupling. A moderate vacancy concentration (Vo‐S‐IO‐6) lowers the protonation barrier by ≈0.2 eV compared to vacancy‐free cubes. FE soars from 7.7% to 60.6%, and the urea rate triples at −0.6 V versus RHE.^[^ ^35^ ^]^ Vacancy engineering is transferable across oxides. In V_O_‐enriched CeO_2_ nanorods, increasing the vacancy population from 0.5% to 1.4% reduces the *OCNO‐to‐*NH‐CO barrier from 0.46 to 0.28 eV. This results in a urea yield of 3.13 µmol cm^−^^2^ h^−^^1^, outperforming several noble‐metal benchmarks.^[^ ^36^ ^]^ In V_O_‐InOOH sheets, a vacancy‐induced surface electron pool can double *OCNO coverage, reduce HER by repelling protons, and increase FE from 26% to 51% while maintaining full activity over 24 h of electrolysis.^[^ ^37^ ^]^

These cases highlight three design rules: i) Pair a single, low‐index facet that orients *OCNO favorably with ii) a vacancy density tuned to remove (not add) ≈0.2 e^−^ from the neighboring cation and iii) keep vacancy fractions below the percolation threshold to prevent over‐stabilization. *CO and throttle desorption. Facet‐defect co‐engineering thus provides a composition‐agnostic route to accelerate the slowest step in the sixteen‐electron cascade without sacrificing selectivity or durability, serving as the foundation for the next wave of oxide and oxy‐hydroxide catalysts that are approaching industrial current densities.

#### 3.1.2 Operational Catalyst Strategies

**Figure 5:**
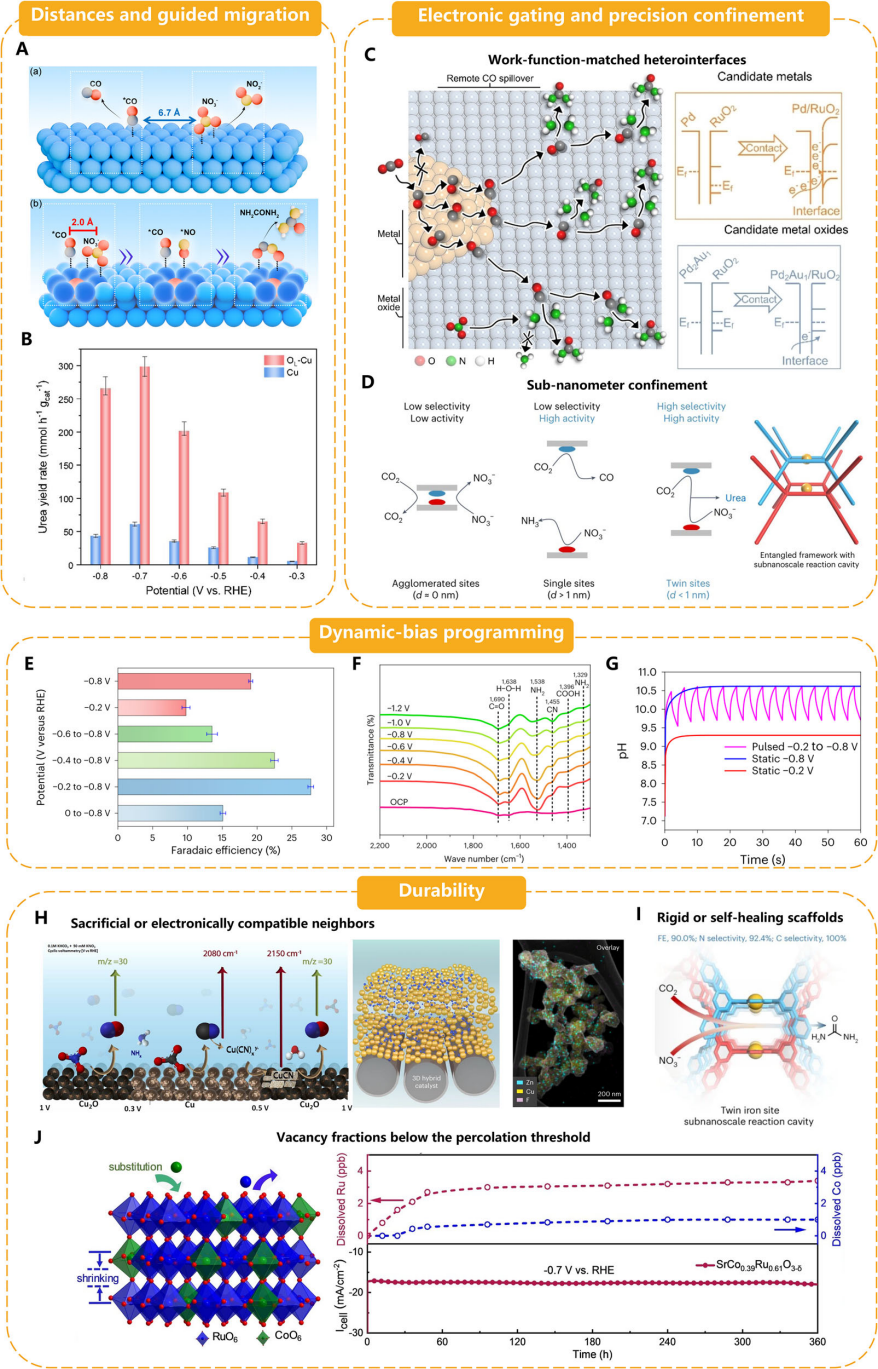
A) Spatial adsorption of CO_2_ and NO_3_^−^ on a typical Cu catalyst and adjacent co‐adsorption of CO_2_ and NO_3_^−^ on electron‐deficient copper sites of the O_L_‐Cu catalyst. The blue, dark blue, red, gray, yellow, and white balls represent Cu, Cu^δ+^, O, C, N, and H atoms, respectively. Reproduced with permission.^[^ ^39^ ^]^ Copyright 2025, American Chemical Society. B) The urea yield rates of the O_L_‐Cu and Cu catalysts at various applied potentials.^[^ ^39^ ^]^ C) Schematic diagram of the design for remote CO* spillover‐improved tandem urea electrosynthesis over the Pd_2_Au_1_/RuO_2_ catalyst. Interfacial electronic configuration analysis of the designed Pd/RuO_2_ with ΔΦ = 0.44 eV and Pd_2_Au_1_/RuO_2_ with ΔΦ = 0.05 eV. Reproduced with permission.^[^ ^34^ ^]^ Copyright 2025, Wiley‐VCH. D) Spatially engineered catalysts for electrocatalytic urea production, utilizing an entangled framework to tailor the cavity with twin metal sites. Reproduced with permission.^[^ ^41^ ^]^ Copyright 2025, Springer Nature. E) FE_urea_ for the Fe‐TPP/CNT‐catalyzed CO_2_/NO_3_^−^ co‐reduction at various pulsed potentials (that is, alternated between −0.6, −0.4, −0.2, 0 and −0.8 V vs RHE) and static potentials (−0.2 and −0.8 V vs RHE). The error bars and the middle of the error bars are the standard deviation and the average of three independent measurements, respectively. Reproduced with permission.^[^ ^44^ ^]^ Copyright 2024, Springer Nature. F) Operando ATR‐SEIRAS spectroscopy measurements for the CO_2_/NO_3_^−^ co‐reduction over Fe‐TPP/CNTs at various potentials from OCP to −1.2 V versus RHE. Reproduced with permission.^[^ ^44^ ^]^ Copyright 2024, Springer Nature. G) Theoretical calculations of pulsed and static pH profile in the boundary layer with distance of 100 µm. Reproduced with permission.^[^ ^44^ ^]^ Copyright 2024, Springer Nature. H) The formation of Cu─C≡N–like species on Cu surfaces during CO_2_ reduction in NO_3_^−^ solution and the scheme, elemental mapping of 3D Cu/Zn hybrid catalysts. Reproduced with permission.^[^ ^45^ ^]^ Copyright 2022, Elsevier. Reproduced with permission.^[^ ^5^ ^]^ Copyright 2023, Springer Nature. I) Scheme of rigid PCOF‐34‐Fe catalyst. Reproduced with permission.^[^ ^41^ ^]^ Copyright 2025, Springer Nature. J) Stability test of Co‐doping O_v_‐rich SrRuO_3_. Reproduced with permission.^[^ ^38^ ^]^ Copyright 2024, Wiley‐VCH.

Operational catalyst strategies encompass methods to choreograph, enhance, and sustain C─N coupling under realistic electrochemical conditions. **Figure** **5** demonstrates four complementary tactics, controlled distances & guided migration, electronic gating & precision confinement, dynamic‐bias programming, and durability engineering, and describes how each contributes to timing, steering, and stabilizing the urea‐forming cascade.

##### Distances and Guided Migration: Ensuring CO Meets NH_2_ Before Protonation

If the adsorbates derived from carbon and nitrogen never come together, linear‐scaling barriers are irrelevant; therefore, careful distance engineering sits alongside electronic tuning in catalyst design. Co‐doped SrRuO_3_ provides a clear example. Introducing 39% Co^3^⁺ ions achieves two goals: i) creating oxygen vacancies that weaken the individual binding of *CO on Co and *NH_2_ on Ru, preventing premature hydrogenation; and ii) contracting the perovskite lattice by ≈0.15 Å, shrinking the Co‐Ru registry to a slip distance of ≈3 Å. Operando synchrotron radiation Fourier transform infrared (SR‐FTIR) detects two intermediates vibrating in phase, indicating co‐adsorption at nearby sites, while density‐functional calculations show the C─N coupling barrier dropping to 0.32 eV. The engineered SrCo_0.39_Ru_0.61_O_3‐δ_ boosts FE to 34.1% and increases the urea rate 7.6‐fold compared to pristine SrRuO_3_.^[^ ^38^ ^]^

Spacing control can also be achieved within a single metal by employing residual lattice oxygen as an electronic wedge. In oxide‐derived Cu nanosheets, sub‐surface O draws electron density from neighboring Cu atoms, resulting in electron‐deficient Cu^δ+^ ensembles. DFT mapping reveals that local depletion reduces the *CO–*NO_3_ separation from 6.7 Å on metallic Cu^0^ to 2.0 Å on Cu^δ+^, falling within the ≈4 Å diffusion length required for spontaneous C─N bond formation. The practical payoff is striking, with a urea production rate of 59.74 µmol cm^−^^2^ h^−^^1^, a steady‐state current density of ≈95 mA cm^−^^2^, and a FE of 31.7% at −0.7 V versus RHE.^[^ ^39^ ^]^ These cases summarize three quantitative rules for “migration engineering”: i) Compress but do not overcompress. Target center‐to‐center distances of 2–4 Å. Shorter gaps (<2 Å) promote µ_2_‐bridge *CO, which poisons the second C─N coupling, a failure mode documented for Ru‐Co and Fe‐Ni dual atoms. ii) Create an electronic gradient. Aliovalent dopants or residual oxygen can skew the charge, causing *CO to diffuse “downhill” toward *NH_2_ instead of hopping randomly. iii) Stabilize the scaffolding. SrCo‐RuO_3_ retains its contracted lattice after 24 h. In contrast, more heavily reduced samples collapse into amorphous oxides, indicating that vacancy fractions or dopant levels that exceed the percolation threshold overwhelm adsorption and erode durability.

Aliovalent doping, controlled oxidation‐reduction cycling, and hetero‐atom infusion in 2D metals provide a composition‐agnostic toolbox for combining *CO and *NH_2_ while preventing hydrogen evolution. The following subsection demonstrates how electronic “gates” and precision cavities build on this spatial foundation to guide the second C─N bond and ensure long‐term selectivity.

##### Electronic Gating and Precision Confinement; Steering Intermediates away from NH_3_/CO and Locking in the Second C─N Bond

Selectivity is contingent upon two delicate equilibrium points: a) *CO must surrender enough electron density to prevent protonation to CO/formate, and b) the surface must maintain the availability of *NH_2_ for the second C─N bond before it is over‐reduced to NH_3_, even after *OCNO has formed. Two complementary design logics address these requirements.

i) Work‐function‐matched heterointerfaces (electronic gating). At oxide‐metal junctions, the local Fermi‐level offset regulates charge accumulation on adsorbates. Alloying Pd with Au in a Pd_2_Au_1_/RuO_2_ nanolaminate reduces the Pd/RuO_2_ work‐function gap from 0.44 to 0.05 eV, reducing excess interfacial charge and loosening. *CO binds. Operando SR‐FTIR shows an intensifying *OCNO band (1628 cm^−^^1^) and fading *COH/*NH_3_ signatures, while electrolysis achieves 75.6% FE, 44.1 µmol cm^−^^2^ h^−^^1^, and a record‐low 18.9 kWh kg_urea_^−^^1^. Mott‐Schottky Bi/BiVO_4_ hybrids use a similar principle: band bending at the metal/semiconductor junction drives electrons toward Bi sites that favor *CO, leaving photogenerated holes to oxidize NO_3_^−^ on BiVO_4_; urea FE trebles relative to bare BiVO_4_ and reaches 12.6% at −0.4 V versus RHE. Tuning ΔΦ to <0.1 eV promotes directional *CO spillover while discouraging HER or CO release.^[^ ^40^ ^]^ ii) Sub‐nanometer confinement (precision cavities). If dual sites are closer than ≈2 Å, *CO forms a µ_2_‐bridge and poisons the final coupling. Gaps larger than ≈5 Å allow fragments to drift apart. Entangled Fe‐porphyrin covalent‐organic framework (COFs) form 3–4 Å cavities with twin Fe‐N_4_ units that align *CO and *NH_2_ vectorially. The rigid organic cage suppresses random migration, blocks bridge‐bonded *CO, and achieves 90% FE with 13.56 µmol cm^−^^2^ h^−^^1^ at −0.5 V versus RHE, which remains the performance benchmark.^[^ ^41^ ^]^ The confinement motif embeds 2 nm γ‐Fe_2_O_3_ particles inside Ni‐HITP MOF pores. The conductive framework stabilizes adjacent Fe(III) centers, which co‐adsorb *COOH and *NO_2_, increasing urea FE to 24% and maintaining activity for 150 h.^[^ ^42^ ^]^

Design rules. a) Flatten interface work‐function mismatches (<0.1 eV) to create an electronic “gate” that times *CO spillover with *NH_2_ availability; b) Fix metal‐metal separations between 2–4 Å in mechanically rigid hosts to prevent µ_2_‐bridge poisoning while guaranteeing encounter probability; c) Integrate operando vibrational probes to verify that *OCNO intensifies while *COH/*NH_3_ signals fade. When combined with the spacing strategies of Co‐doped SrRuO_3_ and Cu^δ+^ sheets, these electronic and geometric locks complete the choreography that shepherds every proton and electron toward the *NH‐CO intermediate and, eventually, urea.

##### Dynamic‐Bias Programming; Synchronizing Voltage with Surface Choreography

In constant‐potential electrolysis, a single fixed bias is required to perform five chemically distinct functions: adsorb nitrate, adsorb CO_2_, forge the first C─N bond, protonate the *OCNO adduct, and prevent hydrogen evolution. These tasks can be decoupled in time by alternating or pulsed waveforms: a mild (or open‐circuit) half‐cycle allows NO_3_^−^ to approach and CO_2_ to dissolve without electrostatic hindrance. In contrast, a stronger cathodic half‐cycle pushes the pre‐adsorbed *CO and *NH_2_ fragments over the C─N‐coupling and protonation barriers. Within the potential range of open circuit potential (OCP) to −0.6 V, in situ attenuated total reflectance Fourier‐transform infrared spectroscopy (ATR‐FTIR) spectroscopy detected peaks at 1419 cm^−^^1^ (C─N bond in urea) and 1695 cm^−^^1^ (C═O bond in *CONO_2_). In situ Raman spectroscopy revealed a peak at 1000 cm^−^^1^ (v_s_(N‐C─N), the characteristic peak of urea) appearing at −0.3 V, confirming the formation of C─N intermediates on CuSiO_x_. In situ Raman also showed that the bridging *CO at 1975 cm^−^^1^ exhibited the highest coverage at −0.2 V, which matched well with the potential for the peak FE of urea.

These results indicate that potential switching (between OCP and −0.2 V) can maintain a high coverage of *CO at high potentials while suppressing the hydrogenation/reduction of *CO.^[^ ^43^ ^]^ Additionally, Attenuated total reflection surface‐enhanced infrared absorption spectroscopy (ATR‐SEIRAS) data revealed a characteristic peak at 1468 cm^−^^1^ (C─N bond), whose intensity increased as the potential shifted negatively, reaching its maximum at the cathodic pulse (−0.8 V) (coinciding with the peak FE potential for urea). This indicates that C─N bond formation is the dominant reaction at high potentials. This process consumes significant H⁺, leading to a local pH increase. Additionally, peaks were detected at 1538 cm^−^^1^ (*NH_2_ peak), 1690 cm^−^^1^ (C═O peak of adsorbed CO_2_), and 1638 cm^−^^1^ (O‐H hydrogen bond of H_2_O molecules), all of which intensified with a positive shift in the applied potential.^[^ ^44^ ^]^

These spectroscopic data confirm the two mechanistic roles of dynamic bias: i) Enhanced local reactant concentration: Achieved through increased local CO_2_ concentration, due to local pH decrease during the anodic pulse (−0.2 V), and the electrostatic attraction concentrating NO_3_^−^. ii) Optimized intermediate coverage via suppression of side reactions: Potential switching suppresses competing reactions: the low‐potential period (−0.2 V) inhibits the HER and consumption of *CO/*NH_2_, while the high‐potential period (−0.8 V) focuses on C─N coupling. It is worth noting that as the coverage of *CO/*NH_2_ increases, it affects the competition for electrons between C─N coupling (urea pathway) and the HER at high potentials, thereby further suppressing the HER at these potentials. Differential electrochemical mass spectrometry (DEMS) data showed that the H_2_ signal intensity during pulsed electrolysis was only 0.4 times that under constant potential, confirming significant HER suppression. Such a “voltage gear‐shifting” is demonstrated in a single‐atom Cu/SiO_2_ nanotube system reported by Qiu et al. Cycling between open circuit and −0.7 V versus RHE neutralizes nitrate repulsion during the OCP window and drives the coupling pulse; the cell sustains 6.41 µmol cm^−^^2^ h^−^^1^ at 79% FE for 24 h.^[^ ^43^ ^]^ A square‐wave program (−0.2 ↔ −0.8 V, 50% duty) applied to Fe‐TPP molecular catalysts achieves homogeneous coverage choreography. When transferred to a PdCu GDE, the protocol increases urea selectivity to 70.4% while reducing electricity use by 41%, bringing the projected cost (with renewable power at US$ 0.03 kWh^−^^1^) to parity with the Bosch–Meiser benchmark.^[^ ^44^ ^]^

The energy‐saving mechanism of pulsed electrolysis primarily manifests in three aspects: i)Enhanced Selectivity. Pulses increase the FE for urea production (e.g., Fe‐TPP: 19% → 28%; PdCu: 40% → 70%), reducing parasitic electron consumption by competing reactions (HER, NH_3_, CO). For instance, in the CuSiO_x_ catalyst, FE increased from 57% under potentiostatic conditions to 79% under pulsed electrolysis at −0.2 V versus RHE, reducing electron loss to competing reactions (e.g., hydrogen evolution or NH_3_ formation). This is consistent with the trend that higher FE correlates with lower urea production costs (e.g., $3.574 kg^−^^1^ at −0.2 V versus $3.679 kg^−^^1^ at −0.6 V). ii) Reduced Overpotential. The average cathode potential during the cathodic pulse shifts positively (e.g., PdCu from −0.6 to −0.4 V vs static operation), lowering the driving voltage (lower average cathode potential). The CuSiO_x_ catalyst demonstrates that pulsed electrolysis at −0.2 V achieves a superior urea yield rate (1606.1 µg h^−^^1^ mg_cat_^−^^1^) with an average cell voltage of 2.5 V, compared to higher voltages (e.g., 3.41 V at −0.6 V) in static operation, directly reducing energy input. iii) Optimized Mass Transport. The anodic pulse promotes reactant diffusion, minimizing concentration polarization losses. The resulting reduction in overpotential is reflected in the lower total urea production cost at optimized potentials (e.g., $3.574 kg^−^^1^ at −0.2 V).^[^ ^43^, ^44^ ^]^

Three quantitative guideposts are common to these and other studies. A voltage amplitude of ≈0.4 V is required to switch between the adsorption and coupling/hydrogenation regimes. Second, a 40–60% duty cycle balances nitrate uptake during the mild phase with urea‐forming pulses, thereby preventing nitrate deficiency. Third, matching pulse widths to the double‐layer recharge time (10^−^^1^–10^0^ s in 0.1 M KHCO_3_/KNO_3_) prevents concentration polarization and limits surface hydrogen build‐up. The catalyst is transformed into a time‐resolved conveyor belt that maintains the lock‐step of *CO and *NH_2_, starves HER, and reduces the kilowatt‐hour price of green urea when dynamic‐bias programming is applied on top of the static levers previously discussed, including dual or relay sites, facet/defect synergy, migration control, and electronic gating.

##### Durability: Designing Surfaces that Maintain Their Choreography

Initial selectivity is insignificant if the active motif restructures or poisons during steady electrolysis. The desirable *CO + *NH_2_ ensemble on metallic Cu slowly rearranges into soluble Cu─C≡N complexes. This growth is visible as new Raman bands at 2080–2150 cm^−^^1^ and is accompanied by measurable Cu loss and NO evolution. The urea rate collapses as the copper surface roughens and the relay of intermediates breaks.^[^ ^45^ ^]^ In Co‐doped SrRuO_3_, the perovskite lattice pins Co and Ru at a fixed 3 Å spacing while oxygen vacancies remain stable, resulting in an unchanged *OCNO signature after 24 h and a FE of ≈34%.^[^ ^38^ ^]^ Rigid organic hosts immobilize twin Fe‐N_4_ centers in 3–4 Å cages, providing 90% FE for 20 h with no crystallinity loss.^[^ ^41^ ^]^ In 3D Zn/Cu GDEs, the oxophilic Zn slowly oxidizes, absorbing cyanide ligands and shielding Cu, ensuring that over 90% of the initial current survives a 32‐h run.^[^ ^5^ ^]^ After 10 h of electrolysis, vacancy‐patched InOOH cubes maintained their 520 cm^−^^1^ Vo band, indicating that moderate (<1%) vacancy densities can withstand oxygen backfill while maintaining 60% FE.^[^ ^37^ ^]^ Collectively, these case studies suggest three practical rules: i) prevent coalescence by locking dual metals in rigid or self‐healing scaffolds, ii) keep vacancy fractions below the percolation threshold to provide electronic flexibility without collapsing, and iii) provide sacrificial or electronically compatible neighbors that sponge up poisons and buffer charge. When combined with periodic, gentle anodic pulses to re‐oxidize the surface cyanide, these material choices extend laboratory breakthroughs to the multi‐day, flow‐cell operation required by techno‐economic models.

### 3.2 Analytical Challenges

**Figure 6:**
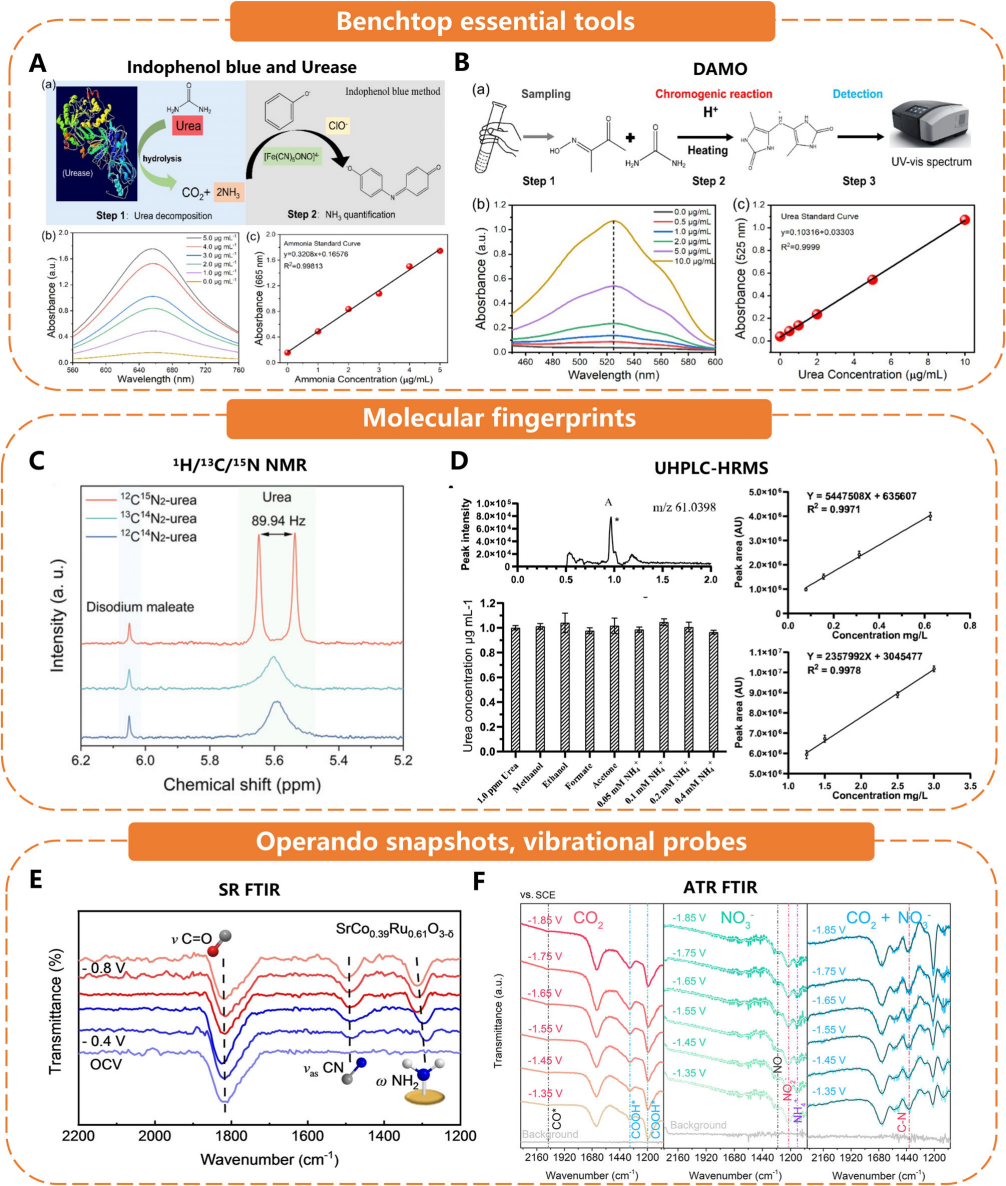
Schematic of the three independent blind round‐robin audits of the urea assay. A) Quantitative urea analysis using the urease method. Reproduced with permission. Reproduced with permission.^[^ ^47^ ^]^ Copyright 2023, Wiley‐VCH. B) Quantitative urea analysis using the DAMO method. Reproduced with permission.^[^ ^47^ ^]^ Copyright 2023, Wiley‐VCH. C) ^1^H‐NMR spectra of non‐isotope‐ and isotope‐labeled urea (10 µg mL^−^^1^). Reproduced with permission.^[^ ^50^ ^]^ Copyright 2024, Nature. D) UHPLC‐HRMS analysis of ^14^N_2_‐urea with 0.1 M KHCO_3_ and 0.1 M KNO_3_ as the sample solvent. Reproduced with permission.^[^ ^47^ ^]^ Copyright 2024, Nature. E) Operando SR‐FTIR spectra of SrCo_0.39_Ru_0.61_O_3−δ_. Reproduced with permission.^[^ ^38^ ^]^ Copyright 2024, Wiley‐VCH. F) In situ ATR‐FTIR spectra of CO_2_, NO_3_^–^_,_ and mixture (NO_3_^–^ + CO_2_) electroreduction over Cu@Zn. Reproduced with permission.^[^ ^49^ ^]^ Copyright 2022, American Chemical Society.

Accurate product analytics serve as the foundation for mechanistic insight and are essential for any credible claim regarding “record‐breaking” reports. In the plasma‐electrochemical co‐reduction of NO_3_^−^/NO_2_^−^ and CO_2_, the target signal is particularly low, typically ≤ 10 ppm of urea, within a complex mixture of NH_3_/NH_4_⁺, NO_2_^−^, NO, CO, HCOO^−^, CH_3_OH, and a significant HER.^[^ ^46^ ^]^ Mis‐assigning even 1% of products as urea can significantly overstate selectivity (FE) by an order of magnitude. Three independent round‐robin blind tests evaluated the community's preferred assays, resulting in a two‐probe workflow resistant to interference. We summarize the findings and incorporate them as the essential analytical foundation for the remainder of this perspective (**Figure** **6**).

#### 3.2.1 Benchtop Essential Tools: Colorimetric and Enzymatic Assays

Indophenol blue and Urease (Limit of detection (LOD) ≈0.52 ppm). Fast, cheap, and ideal for high‐throughput screening, but strongly perturbed by pH drift, enzyme aging, NH_3_/NH_4_⁺ crossover, and trace NO_2_^−^. Rigorous blanks, nitrite scavengers, and a second orthogonal assay are mandatory.

Diacetylmonoxime (DAMO, LOD ≈0.14 ppm). Less sensitive to ammonium; however, > 1 mM NO_2_^−^ quenches the chromophore. The field now favors the DAMO‐TSC variant (thiourea/Fe^3^⁺), which destroys nitrite in situ and keeps the error below ±3 % up to 30 ppm NO_2_^−^.

#### 3.2.2 Molecular Fingerprints: High‐Resolution Chromatography and NMR

##### High‐Resolution Analytical Techniques

^1^H/^13^C/^15^N nuclear magnetic resonance (NMR) (LOD ≈ 0.55 ppm at 600 MHz). Quantifies urea and by‐products simultaneously and clearly establishes C─N coupling using dual‐labeled ^13^CO_2_/^15^NO_3_^−^. Disadvantages include water‐suppression pulse program requirements, carbonate overlap in KHCO_3_, and lengthy acquisition times when titers fall below five ppm.

Ultra‐high‐performance liquid chromatography‐high resolution mass spectrometry (UHPLC‐HRMS) (Orbitrap/Time of flight mass spectrometer (TOF), amide‐hydrophilic interaction liquid chromatography (HILIC) column, LOD ≈0.42 ppm). Provides sub‐ppm accuracy in electrolytes containing 10 mM CH_3_OH, HCOO^−^, NH_4_⁺, or NO_2_^−^, which are matrices that impair color tests, in 0.1 M KHCO_3_/0.1 M KNO_3_. Injection losses are corrected for by an internal standard (thiourea), and co‐elution artifacts are removed using exact‐mass detection (m/z 61.0398 ± 3 ppm).^[^ ^47^ ^]^

##### In Situ Vibrational Spectroscopy

Operando snapshots, vibrational probes. ATR‐FTIR, SR‐FTIR, polarisation modulation infrared reflection absorption spectroscopy (PM‐IRRAS), and surface‐enhanced Raman scattering (SERS) do not provide absolute concentrations; however, they confirm the formation of a genuine C─N bond on the catalyst surface and differentiate other products from urea.

PM‐IRRAS/ATR‐SEIRAS. IRRAS is a qualitative detection method that selectively detects adsorption intermediates according to selection rules, but is sensitive to the matrix. In particular, ATR‐SEIRAS is a surface‐sensitive, in situ, label‐free spectroelectrochemical technique. Time‐resolved in situ ATR‐SEIRAS showed decreasing peaks on RP‐CuAu at 1455 cm^−^^1^ (urea v_as_(NCN)), 1305 cm^−^^1^ (intermediate _v_(CN)), and 1720 cm^−^^1^ (amide I), indicating continuous COOH/*NH_2_ coupling intermediate formation. Concurrently, the amide C═O peak (1760 cm^−^^1^) intensified with reaction time, confirming continuous urea generation during electrochemical C─N coupling.^[^ ^48^ ^]^ In situ IRRAS on Zn/Cu catalyst detected the *COOHNH_2_ dimer C═O peak (1694 cm^−^^1^) at −0.3 V versus RHE. Its blue‐shift indicates O─(C═O) coordination with catalyst metal ions. The concurrent OCO band (1403 cm^−^^1^) confirms *CO_2_NH_2_ presence, evidencing the key CO_2_NH_2_ → COOHNH_2_ protonation step in urea synthesis.^[^ ^5^ ^]^

SERS. A semi‐quantitative method with high sensitivity. Adsorbed intermediates can be identified according to selection rules, and specific urea‐sensitive enzymes can be fixed on the substrate to improve selectivity. SERS tracked spill‑over CO* on Pd_2_Au_1_/RuO_2_ during pulsed electrolysis.^[^ ^34^ ^]^ In situ surface‐enhanced Raman spectroscopy (SERS) sensitively detected intermediates on the Zn/Cu hybrid catalyst and single‐component copper surface under urea synthesis conditions, revealing that the M–OCONH_2_ (*CO_2_NH_2_) signal (334–337 cm^−^^1^) was only detected on the hybrid catalyst, providing evidence for the hypothesis that zinc may participate in the formation of the required *CO_2_NH_2_ intermediate.^[^ ^5^ ^]^

SR‐FTIR/ATR‐FTIR. A common semi‐quantitative method for detecting urea. It is highly sensitive and compatible with various substrates. However, it detects the product in the liquid phase interface rather than the adsorbed product. Diagnostic bands include the C═O stretch (≈1815 cm^−^^1^), C─N stretch (≈1415 cm^−^^1^), and NH_2_ wag (≈1310 cm^−^^1^).^[^ ^38^ ^]^ Time‐resolved SR‐FTIR has even caught the fleeting OCNO intermediate at 1628 cm^−^^1^ on Cu‐Zn GDEs.^[^ ^49^ ^] 15^N isotope‐labelled SR‐FTIR spectra further confirm the enhanced C─N coupling activity on O_L_‐Cu by showing red shifts in the peak positions of nitrogen‐containing intermediates (e.g., δ(−NH_2_), 1099 cm^−^^1^).^[^ ^39^ ^]^

#### 3.2.3 A Pragmatic Recipe, Implementing the “Two‐Probe” Rule

Based on three independent blind round‐robin audits of the field's most common assays, we recommend the following minimal analytical workflow for unambiguous urea quantification and C─N coupling verification:

Initially, an analysis of each sample should be conducted using two orthogonal methods: a bulk, high‐throughput assay (e.g., the DAMO‐TSC diacetylmonoxime test or the urease/indophenol assay) and a structure‐specific technique (e.g., UHPLC–HRMS/HPLC–MS or dual‐label ^13^C/^15^N NMR). This dual approach balances the sensitivity of colorimetric tests to NO_2_^−^/NH_3_ interference with the chemical specificity of chromatographic or isotope‐resolved spectroscopic detection.

Secondly, all measurements must be accompanied by full calibration curves in the exact electrolyte composition used (e.g., 0.1 M KHCO_3_ + 0.1 M KNO_3_), as well as spike‐recovery controls at low concentrations (e.g., 0.4 ppm and 0.8 ppm urea) and catalyst‐free electrolyte blanks processed in parallel. These checks measure matrix effects, sample handling losses, and background contamination.

Thirdly, dual‐isotope tracer experiments (^13^CO_2_ with ^15^NO_3_^−^) should be conducted whenever feasible to definitively establish that the nitrogen and carbon atoms in the detected urea are derived from the intended feedstocks rather than adventitious sources. Claims of improved urea production and C─N coupling can only be substantiated by rigors, two‐probe strategies that combine a bulk enzymatic or colorimetric test with a structure‐specific molecular fingerprint, which is validated by calibration, blanks, and isotopic tracing.

### 3.3 Urea Versus Other C─N‐Containing Molecules

Selective electro‐synthesis of urea proceeds through a tightly choreographed, sixteen‐electron cascade in which surface‐adsorbed *CO/*CHO fragments must couple with *NH_2_ species at matched rates. Any kinetic or micro‐environmental imbalance fractures this synchrony and opens lower‐energy pathways to smaller amines and amides. When *NH_2_ fragments accumulate more rapidly than carbon‐bound adsorbates, an over‐supply often observed on Bi‐rich or Fe‐rich facets, they are over‐hydrogenated to NH_3_ or attack dissolved CO_2_ to yield formamide or methylamine. A converse surplus of *CO/*CHO leads to premature CO or formate release before C─N coupling can occur. Even with stoichiometric surface pools, selectivity is lost if the *OCNO → *NH–CO protonation barrier remains above ≈0.4 eV; under such conditions *OCNO desorbs or rearranges to carbamates and short amides instead of progressing to urea. These vulnerabilities are amplified by steep pH gradients in gas‐diffusion layers, where local alkalinisation stabilises *NH_2_OH, the immediate precursor to alkylamines, whereas transient acidic pockets protonate *NO to NH_3_. Finally, a static cathodic bias strong enough to drive C─N coupling will also accelerate terminal hydrogenations, redirecting partially hydrogenated intermediates toward NH_3_ or CH_3_NH_2_. Operando spectroscopy and ab initio kinetics now confirm each of these branch points.^[^ ^51^ ^]^

Once the preferred urea pathway is compromised, the reaction is diverted toward two dominant product families: primary and secondary amines, methyl‐, dimethyl‐, ethyl‐amine, with occasional hydrazine or hydroxylamine, and simple amides or carbamates such as formamide, acetamide, and carbamate esters. These species exploit the same nucleophilic‐carbonyl chemistry that underpins classical urease‐indophenol and DAMO‐TSC assays and therefore display identical chromophores.^[^ ^52^ ^]^ Three experimentally reproducible scenarios illustrate the drift: rapid *NH_2_OH build‐up that outpaces downstream hydrogenation on CoPC─NH_2_/CNT cathodes (13% FE to methylamine), enrichment of *CO/*CHO relative to *OCNO on Cu triple‐phase electrodes that steers CO_2_/NH_3_ feeds toward formamide and acetamide, and persistence of ketene‐ or cyanide‐like intermediates on low‐coordination Cu sites that favour formamide over urea.^[^ ^53^ ^]^ Recent reports on CuO_x_/BiO_x_ nanocomposites have confirmed formamide formation via *CHO–*NH_2_ coupling, with operando SR‐FTIR and DFT attributing this pathway to selective stabilization of both intermediates under moderate alkaline bias and dual‐site catalysis.^[^ ^54^ ^]^ Separately, acetamide was observed to emerge from aldoxime intermediates on Cu nanoparticles under gas‐diffusion‐fed alkaline electrolysis, where acetaldehyde and NH_2_OH undergo field‐driven dehydration and hydrolysis cascades.^[^ ^55^ ^]^ If left unchecked, these off‐path products can inflate apparent urea efficiencies by an order of magnitude when true urea titres drop below ≈10 ppm.

**Figure 7:**
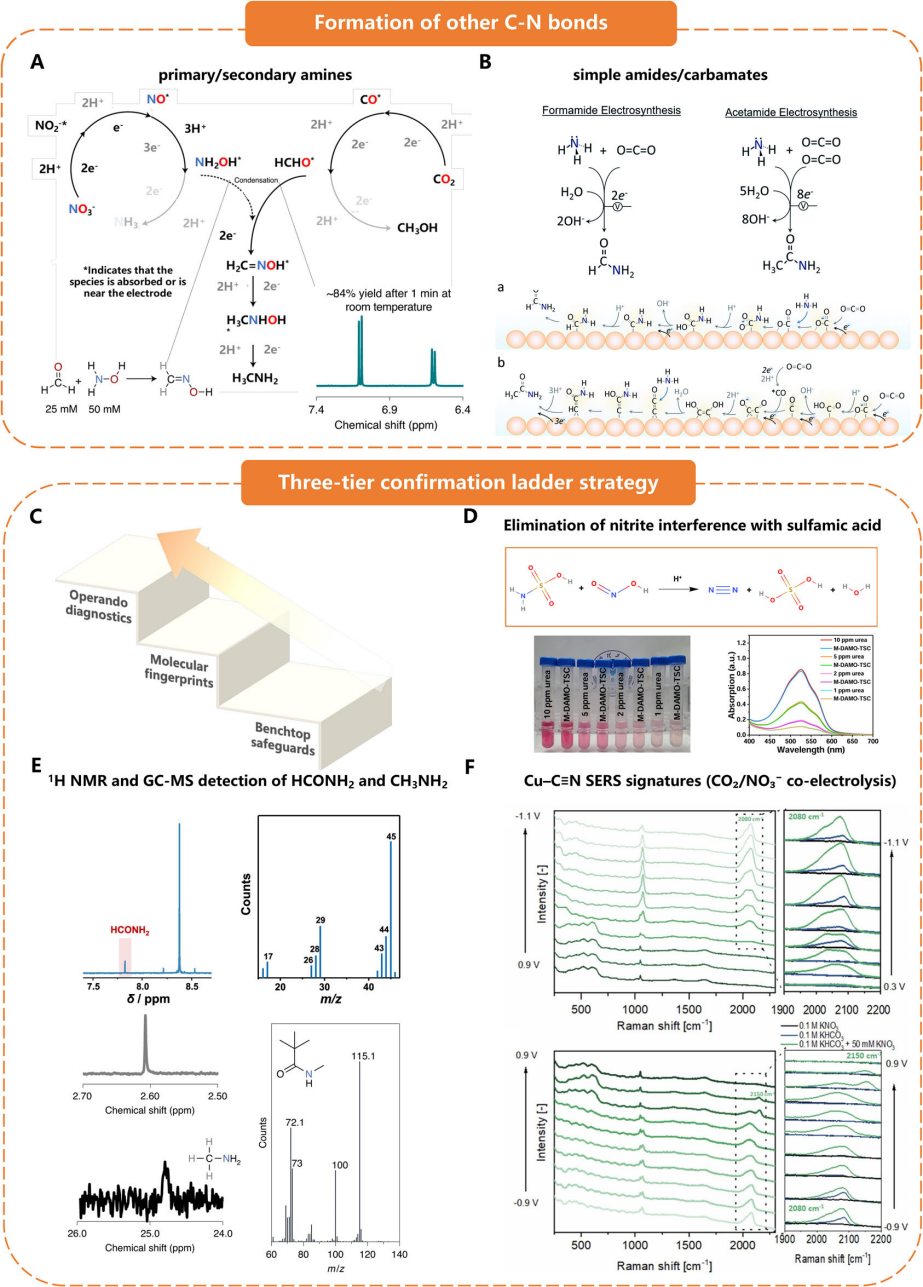
Scenarios for other C─N bonds and methods of differentiation. A) The proposed reaction pathway of the eight‐step cascade electrosynthesis of methylamine from CO_2_ and NO_3_^−^ catalysed by CoPC─NH_2_/CNT. Reproduced with permission.^[^ ^53a^ ^]^ Copyright 2021, Springer Nature. B) Plausible surface reaction pathways in the electrosynthetic process of formamide and acetamide generation. Reproduced with permission.^[^ ^53b^ ^]^ Copyright 2022, The Royal Society of Chemistry. C) The proposed three‐tier confirmation ladder strategy. D) An improved DAMO‐TSC approach. Reproduced with permission.^[^ ^56^ ^]^ Copyright 2023, Wiley‐VCH. E) ^1^H NMR and GC‐MS detection of formamide and ^1^H NMR, ^13^C NMR, and GC–MS detection of methylamine. Reproduced with permission.^[^ ^53a^ ^]^ Copyright 2021, Springer Nature. Reproduced with permission.^[^ ^53c^ ^]^ Copyright 2022, American Chemical Society. F) In situ SERS spectra recorded during a reductive scan from 0.9 V → − 0.9 V and a scan in the positive direction from − 0.9 to 0.9 V versus RHE. Both Raman bands, at ≈2080 and ≈2150 cm^−1^, prove that Cu─C≡N–like species are formed. Reproduced with permission.^[^ ^45^ ^]^ Copyright 2022, Elsevier.

To prevent such misassignments and to quantify genuine urea production, we propose a three‐tier confirmation ladder in which each analytical tier couples a targeted diagnostic with its corresponding operational control (**Figure** **7**). Tier 1 employs rapid colour tests to flag gross deviations in product speciation; Tier 2 deploys molecular fingerprints (^1^H‐NMR, UHPLC‐HRMS) to resolve urea from co‐eluting amines and amides; Tier 3 integrates operando vibrational spectroscopy with isotopic tracing to validate mechanistic assignments under true reaction conditions. Applied in concert, this ladder selectively distinguishes and suppresses amine/amide formation, ensuring that reported FEs faithfully reflect urea synthesis rather than chromophoric impostors.

#### 3.3.1 Benchtop Safeguards

Conduct a rapid orthogonal dip test after performing the standard urease‐indophenol or DAMO‐TSC assay. Primary amines can be detected by nitroprusside or Nessler's reagent. Formaldehyde or formamide can be detected by a fluoride‐quenched Nash assay, and a 20 mM sulphite can eliminate nitrite‐driven artifacts quench.^[^ ^56^ ^]^ To correct residual cross‐signal, use an internal standard, such as thiourea. To reduce by‐products in situ, we recommend fine‐tuning the cathodic potential (e.g., avoiding potentials below −0.9 vs RHE that drive NH_2_OH build‐up), buffering the electrolyte at neutral to mildly alkaline pH, and employing catalysts, such as Cu‐Zn dual‐site alloys, that rapidly hydrogenate *NH_2_ before it can divert to amines or amides.^[^ ^5^ ^]^

#### 3.3.2 Molecular Fingerprints

Each data set must include a structure‐specific probe to validate urea. UHPLC‐HRMS on an amide‐HILIC column resolves m/z 45.0215 (formamide) and 59.0370 (acetamide) from urea at 61.0398 within four minutes, even in electrolytes of 0.1 M KHCO_3_/0.1 M KNO_3_.^[^ ^47^ ^]^ Formamide has an 8.1 ppm singlet/163 ppm carbonyl pair, while acetamide has a 2.0 ppm methyl triplet, which are not present in urea spectra. When ultra‐trace amines are suspected, gas chromatography‐mass spectrometry (GC‐MS) after pivalate derivatization confirms methylamine at femtomolar levels. Tracking the carbon balance (formate, ketene, or CO partial currents) provides an early warning of amide drift.

#### 3.3.3 Operando Diagnostics

In situ vibrational spectroscopy identifies the exact moment and conditions when off‐path products emerge. Amide C═O stretches (≈1680 cm^−^^1^) and N‐H bends (≈1550 cm^−^^1^) are red‐shifted compared to urea's diagnostic bands at 1815/1310 cm^−^^1^, while primary amines show NH_2_ scissoring vibrations near 1600 cm^−^^1^.^[^ ^53c^ ^]^ SERS identifies Cu─C≡N surface complexes based on their 2080–2150 cm^−^^1^ peaks.^[^ ^45^ ^]^ Recording these signatures during potential sweeps or nitrate‐pulsed feeds confirms the formation of side products. It pinpoints the reaction window, guiding adjustments to electrolyte composition and catalyst design to steer intermediates back toward the OCNO and into the urea pathway.

Using a multi‐tiered approach that includes benchtop dip‐tests, molecular fingerprinting, and operando spectroscopy, researchers can accurately quantify and correct for amine and amide impurities, resulting in robust and reproducible urea selectivities. Although dual‐isotope labelling (^13^C/^15^N) is the definitive proof of carbon and nitrogen provenance, its high cost, complexity, and limited accessibility make it unsuitable for routine screening. To identify urea without relying solely on isotopic tracers, complementary strategies such as carbon‐balance checks (tracking formate, ketene, or CO partial currents), internal‐standard corrections in colorimetric assays, and rapid UHPLC‐HRMS/NMR validation provide a reliable and accessible pathway.

#### 3.3.4 Strategies for Suppressing Amine/Amide Formation

**Figure 8:**
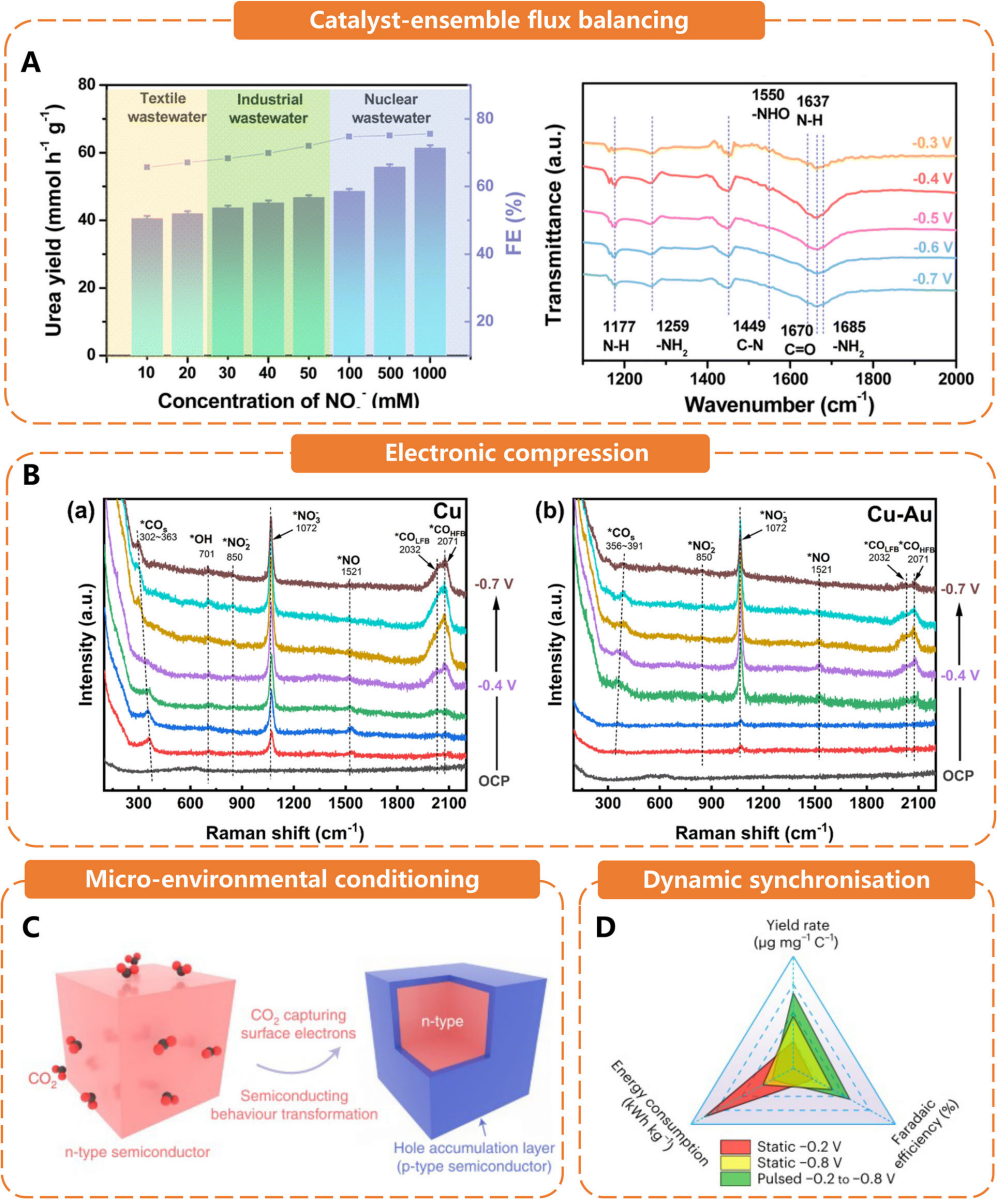
Strategies for suppressing amine/amide formation. A) Yield and FE of urea in different concentration nitrate sources (contaminated textile wastewater, industrial wastewater, and liquid nuclear wastes). In situ FTIR spectra of Ru–Cu_9_Bi/CNT at different potentials. Reproduced with permission.^[^ ^57^ ^]^ Copyright 2024, The Royal Society of Chemistry. B) In situ Raman spectra of Cu and Cu–Au in a CO_2_‐saturated electrolyte solution at potentials from the open circuit state to −0.7 V (the growth step of cathode potential is −0.1 V). Reproduced with permission.^[^ ^58^ ^]^ Copyright 2025, The Royal Society of Chemistry. C) Schematic illustration of the n‐p transformation process in a semiconductor type. The left image is the n‐type In(OH)_3_, while the right image displays the generation of a surface p‐type layer on In(OH)_3_ induced by CO_2_ capture. Reproduced with permission.^[^ ^59^ ^]^ Copyright 2021, Springer Nature. D) Comparing the FE, yield rate and energy consumption of urea at pulsed potentials (that is, alternated between −0.2 and −0.8 vs RHE) and a static potential of −0.2 and −0.8 V versus RHE. Reproduced with permission.^[^ ^44^ ^]^ Copyright 2024, Springer Nature.

Achieving nitrogen‑specific selectivity exceeding 90%, rather than merely raising the gross urea current density, has emerged as the decisive techno‐economic lever for electrochemical CO_2_/NO_x_‐to‐urea synthesis. As shown in **Figure** **8**, four complementary control dimensions now crystallise from the recent literature: i) catalyst‑ensemble flux balancing, ii) electronic compression of the *OCNO proton‑coupling barrier, iii) micro‑environmental conditioning, and iv) dynamic synchronisation of adsorption and coupling events.

Selective flux balancing is demonstrated most clearly in relay‑site catalysts that couple electron‑rich Cu with oxophilic or nitridophilic neighbours. A Ru‑doped Cu–Bi nanocomposite maintains commensurate surface coverages of *CO/*CHO and *NH_2_ across a ten‑fold swing in nitrate concentration, thereby sustaining a 75.6% FE to urea at 150 mA cm^−^^2^ while liquid‑phase amine signals fall below the UHPLC‑HRMS detection threshold.^[^ ^57^ ^]^ By eliminating the kinetic mismatch between the carbon‑bound (*CO/*CHO) and nitrogen‑bound (*NH_2_) surface pools that would otherwise trigger over‑hydrogenation of *NH_2_ or premature CO/formate desorption, the material closes the first mechanistic escape hatch in the urea cascade.

Electronic barrier compression tackles the rearrangement of the *OCNO intermediate. Trace substitution of Au into Cu(111) lowers the *OCNO → *NH–CO proton‑coupling barrier from ≈0.44 to 0.15 eV; carbamate and short‑amide yields subsequently drop below three per cent of the nitrogen product slate.^[^ ^58^ ^]^ Density‑functional calculations attribute the effect to back‑donation that stabilises the transition state, while operando ATR‑FTIR confirms the disappearance of the 1680 cm^−^^1^ amide C═O stretch.

Locally conditioned boundary layers now provide a demonstrable route to curbing amide drift. Two recent flow‐cell studies illustrate the principle. Lv et al. employed a 50 µm cation‐exchange membrane that collapsed the catholyte film to < 100 µm and confined proton transport to the catalyst interface; the resulting In(OH)_3_ GDE sustained 53% FE to urea while the amide C═O stretch (≈ 1680 cm^−^^1^) was suppressed to the noise level at 50 mA cm^−^^2^.^[^ ^59^ ^]^ Building on this architecture, Hu et al. combined a thin bipolar membrane with millisecond pulsed bias, attenuating carbonate build‐up and holding the interfacial pH within ±0.5 units; at 150 mA cm^−^^2^ the urea‐to‐amide ratio doubled relative to a conventional flow‐by cell while operando ATR‐FTIR again showed no detectable amide band.^[^ ^44^ ^]^ Collectively, these studies demonstrate that rigorous boundary‐layer control, achieved by compressing the liquid film, pinning proton or hydroxide fluxes at the catalyst–membrane interface, and dynamically moderating the local pH, provides an effective and scalable means of suppressing amide and amine drift.

Temporal programming breaks the fixed‑bias compromise between adsorption and coupling. Millisecond square‑wave operation (−0.2 ↔ −0.8 V vs RHE, 50% duty) shortens the lifetime of *NH_2_OH and *CHO, cutting methyl‑amine formation by an order of magnitude while pushing urea FE past seventy per cent at 300 mA cm^−^^2^.^[^ ^44^ ^]^ When the same study then coupled duty‑cycled bias with 200 ms CO_2_ “breaths” every ten seconds, carbonate was flushed and formamide drift was arrested on Cu/BiO_x_ nanocomposites.^[^ ^54^ ^]^

### 3.4 Electrolyzer Architecture and Design

**Figure 9:**
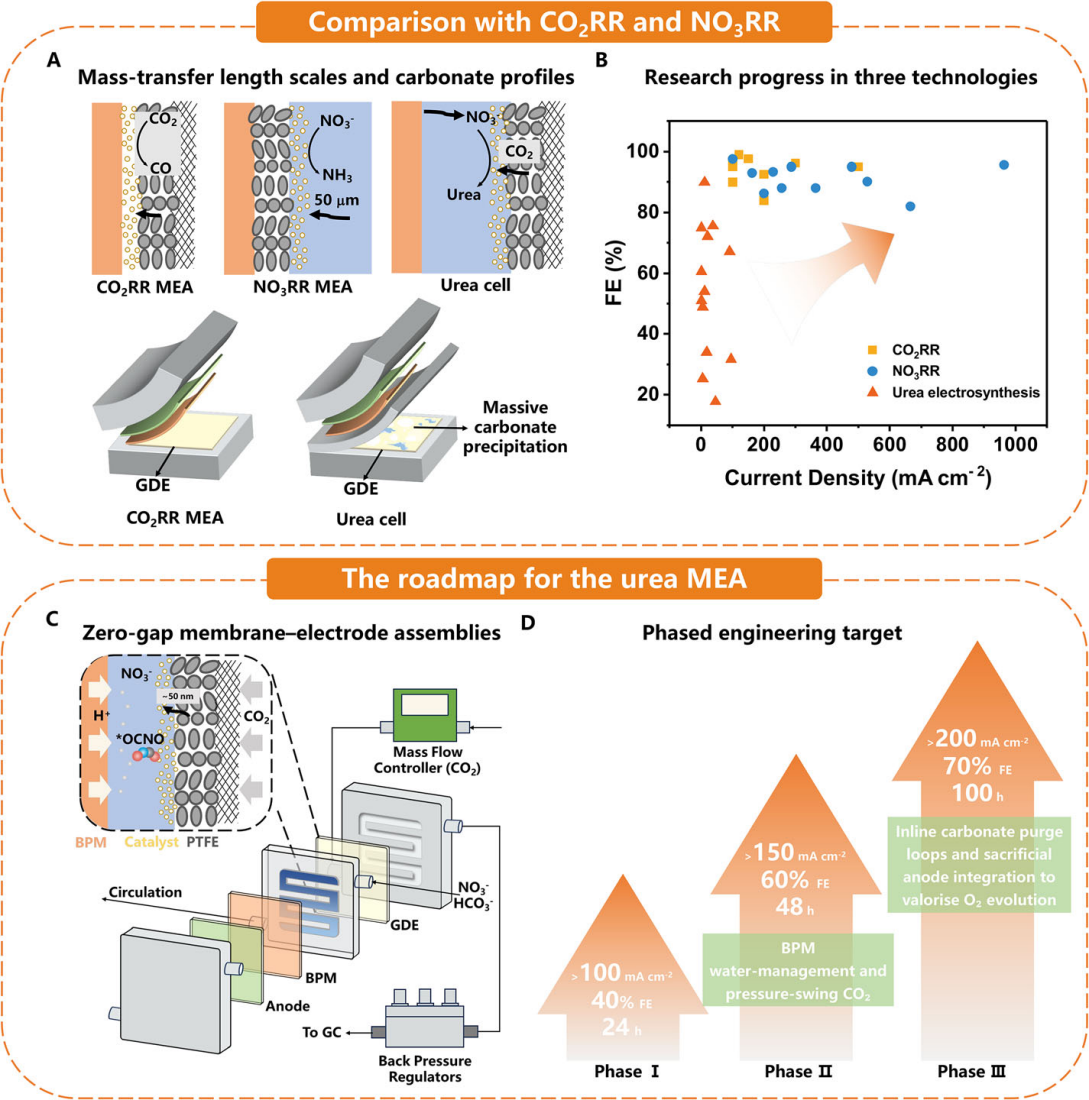
A) Mass transfer length scales and carbonate profiles in CO_2_RR, NO_3_RR, and urea cells. B) Current density versus FE progress of the three technologies. C) Exploded view of a urea MEA stack. D) A phased engineering target for the urea MEA.

It is necessary to make more than incremental adjustments to translate molecular‐level catalyst innovations into a scalable and viable electrolyzer (**Figure** **9**). The electrosynthesis of urea from plasma‐generated nitrate (NO_3_^−^) and CO_2_ has inherent complexity due to its 16‐electron and 18‐proton reaction cascade. To achieve industrially relevant currents (>100 mA cm^−^^2^) and selectivities (>70% FE), advanced reactor architectures are needed that address mass transfer, electrolyte management, interface control, and process stability challenges.

#### 3.4.1 Challenges and Opportunities from Related Technologies; What CO_2_RR and NO_3_RR Teach Us, and Where Urea is Fundamentally Different

Electrolyzers provide a clearly defined starting point for single‐substrate reactions. In less than five years, gas‐fed CO_2_ cells transitioned from H‐cells (<10 mA cm^−^^2^) to hydrophobic GDE flow plates (>250 mA cm^−^^2^). This transition was primarily achieved by eliminating centimeter‐thick liquid films and establishing a stable triple‐phase boundary between gas, liquid, and solid.^[^ ^60^ ^]^ Using pressurized feeds and zero‐gap MEAs, nitrate‐to‐ammonia stacks can achieve FEs of over 90%.^[^ ^61^ ^]^ These accomplishments establish three design principles that are immediately transferable to urea electrosynthesis: i) Reduce the diffusion path for gaseous reactants to micrometers. ii) Keep the catalyst layer dry and conductive for weeks rather than hours. iii) Combine mass‐transfer engineering with local pH control to reduce hydrogen evolution.

However, urea synthesis introduces two additional layers of complexity not encountered by CO_2_RR or NO_3_RR in isolation: (i) Dual‐feed synchronization. The catalytic surface must contain both *CO‐derived and *NH_2_‐derived fragments in the proper stoichiometry. When CO_2_ is depleted, *NH_2_ over‐hydrogenates to NH_3_, while if nitrate is scarce, *CO drifts to CO/formate. The residence times and boundary layer thicknesses of the two feeds must match within ±10%, which is a much tighter tolerance than for single‐substrate devices. (ii) Carbonate management during alkaline drift. Proton consumption by the 16‐electron cascade raises the pH near the surface by 2–3 units, causing K_2_CO_3_ to precipitate and eventually block the GDE. Because nitrate reduction consumes OH^−^ concurrently, the carbonate load is 50–70% higher than in CO_2_‐only cells with comparable current densities.

This “alkaline drift” is not yet documented for urea flow‐cells, so we extrapolated from the best‐resolved CO_2_RR literature to warn that carbonate precipitation will become a first‐order degradation pathway once urea devices reach current densities above ≈100 mA cm^−^^2^. Operando wide‐angle X‐ray scattering on a Cu GDE showed KHCO_3_ crystallising linearly at 1.4 µmol cm^−^^2^ h^−^^1^ under 100 mA cm^−^^2^, with CO FE plunging from 93% to <20% once ≈35% of the microporous layer was occluded.^[^ ^62^ ^]^ In an independent Ag‐GDE study the double‐layer capacitance, used as a proxy for electrolyte flooding, reached its sigmoidal inflection after 3.8 C cm^−^^2^ (≈ 1 h at 100 mA cm^−^^2^); CO selectivity halved, and the total current fell by 30%.^[^ ^63^ ^]^ Cathode pressurisation to 10 bar suppressed carbonate nucleation by over 70%, extending high‐selectivity operation at 300 mA cm^−^^2^ from 1.5 h to more than 6 h while adding <2% to stack power demand.^[^ ^60^ ^]^ A reverse‐bias bipolar‐membrane MEA eliminated carbonate formation altogether: the carbon‐cross‐over coefficient fell to zero and near‐quantitative CO selectivity persisted for 100 h at 500 mA cm^−^^2^.^[^ ^64^ ^]^ Finally, a hydrophobic‐gradient microporous layer (MPL) (contact angle 118 → 142° across 50 µm) cut the accumulated salt mass by 70% after 10 h at 150 mA cm^−^^2^ and limited the accompanying voltage drift to <50 mV.^[^ ^65^ ^]^

Because nitrate‐to‐urea releases four times more OH^−^ than the two‐electron CO_2_→CO pathway, stoichiometric scaling of the upper‐bound CO_2_RR rate (1.4–1.6 µmol cm^−^^2^ h^−^^1^) predicts a carbonate flux of roughly 2–3 µmol cm^−^^2^ h^−^^1^ at 100 mA cm^−^^2^ in a urea cell, a figure that aligns with continuum modelling of alkaline CO_2_/NO_3_^−^ co‐electrolysis.^[^ ^66^ ^]^ Once dual‐feed reactors achieve practical current densities, the onset of carbonate fouling can be expected within the first hour of operation, and selectivity decay will track the same 30–40% pore‐blocking threshold documented for CO_2_RR.

The very fact that two reactants must meet at a single interface opens design space unavailable to simpler chemistries: dual‐channel flow plates that tune the CO_2_∶NO_3_^−^ flux ratio on‐the‐fly; pulsed‐pressure operation where a brief CO_2_ “breath” scours carbonate before a nitrate‐rich pulse restores the pH; and sequential MEAs that reduce NO_3_^−^ upstream, then feed nitrite‐rich electrolyte directly into a downstream CO_2_‐coupling cathode. Urea electrosynthesis requires simultaneous control of two gaseous feeds, mitigation of amplified carbonate fouling, and synchronization with an upstream plasma module that delivers nitrate at fluctuating rates with residual oxygen. The following sections translate these demands into specific hardware options and operating protocols.

#### 3.4.2 GDEs: Mass Transport Innovations

According to the experience gained in CO_2_‐to‐CO and nitrate‐to‐NH_3_ electrolyzers, the GDE is the most effective lever for transitioning from milli‐ to ampere‐scale current densities. The GDE must, however, sustain a heavier carbonate burden while regulating a dual gas/liquid feed for urea electrosynthesis. Three intertwined design parameters dominate performance. i) A hydrophobic MPL. A PTFE‐rich MPL with a static contact angle > 115° prevents liquid water from entering gas pores for over 12 h at 100 mA cm^−^^2^ and delays breakthrough, reducing carbonate crystallization. Experimental data confirm that at 300 mA cm^−^^2^, electrolyte leakage rates, an established proxy for carbonate accumulation, are suppressed to <10% of untreated MPL baselines, thus extending stable electrolyzer operation by 50‐fold.^[^ ^67^ ^]^ At optimal PTFE loadings (30–50 wt%), the pore size distribution is bimodal, with pores ranging from 0.1 to 0.5 µm pinning the liquid‐gas interface and pores ranging from 2 to 5 µm venting CO_2_. To match the same lifetime in CO_2_RR, the breakthrough time scales linearly with the product of contact angle and MPL thickness. For urea cells with higher local alkalinity, doubling the thickness to 60–80 µm or adding a graded‐hydrophobicity overlayer is required.^[^ ^68^ ^]^ ii) Pressure‐assisted cathode operation. Running the gas channel at 2–3 bar increases CO_2_ solubility from ≈33 mM to over 90 mM at 25 °C, shifting the carbonate precipitation front downstream of the catalyst layer.^[^ ^69^ ^]^

Cathode pressurization triggers reverse osmosis (RO), driving water from cathode to anode. This reduces cathode water accumulation while suppressing electro‐osmosis‐induced ion loss and local pH fluctuations. At 6 atm, Ag/AEI‐C electrodes maintained FE_CO_ >90% for 12 h without decay (vs 6 h at 1 atm; 4 h with high‐K⁺ anolyte where FE_CO_ plummeted from 94.5% to 63.1%). No carbonate‐deposition blockage occurred under pressure.^[^ ^70^ ^]^ In nitrate‐to‐NH_3_ stacks, this strategy extends stable operation from 10 to >40 h. Early urea prototypes see a three‐fold reduction in carbonate‐induced overpotential when a 2.5‐bar CO_2_ blanket is applied. Transient pressure‐swing cycles (200 ms CO_2_ “breaths”) effectively flush nascent crystals before they bridge pores, with a negligible energy penalty (<1% of stack power). iii) Binder and catalyst layer mechanics. Conventional Nafion binders are brittle and cause shear‐induced delamination within hours of high flow. Replacing Nafion with perfluoro‐alkylated styrene ionomers increases peel strength from 0.12 to 0.45 MPa, accommodates 2–3 % volumetric swelling during carbonate deposition, and maintains electronic contact for 96 h at 150 mA cm^−^^2^ in nitrate cells. Scanning electron microscope (SEM) analysis showed no carbonate deposition on the 5 wt% PTFE Ag cathode after a 6 h durability test (200 mA cm^−^^2^, 2 M KOH). This is attributed to PTFE's high hydrophobicity (contact angle > 85°) and the stable microstructure formed during 250 °C annealing, which suppresses carbonate nucleation.^[^ ^71^ ^]^ Hot‐spray or ultrasonic‐spray techniques create interlocked fibrils that dissipate hydrodynamic stress, a crucial upgrade for turbulent flows (Re > 1000) in industrial urea stacks. iv) Acid‐humidified cathode feed stream.

Feed management innovations reveal that introducing trace volatile acids into the CO_2_ feed stream effectively prevents (bi)carbonate precipitation and sustains continuous CO_2_ reduction for up to 4500 h. Quantitative ion chromatography demonstrates identical K⁺ crossover rates (≈0.09 µmol K⁺ h^−^^1^ in 1‐cm^2^ electrolyzers) under both H_2_O‐ and acid‐humidification, yet H_2_O‐humidified systems fail within 960 h due to salt blockage at a KHCO_3_ removal rate of 0.09 µmol K⁺ h^−^^1^. Conversely, 0.05 M HCl humidification achieves a sustained K⁺ removal rate of 0.5 µmol h^−1^ over 2000 h with no observable salt crystals in flow channels. In scaled 100‐cm^2^ flow cells, severe salt precipitation limits H_2_O‐humidified CO_2_ stability to ≈80 h, whereas HNO_3_ humidification maintains <19 µV h^−^^1^ voltage degradation and 80–90% CO FE over 4500 h. Operando imaging and SEM confirm salt‐free flow channels under acid humidification, with effluent analysis verifying soluble potassium salts (e.g., KCl), demonstrating that acid vapor enhances solubility to convert carbonates into ionic species for convective removal. Furthermore, extending this acid‐humidified CO_2_ approach to ZnO, Bi_2_O_3_, and Cu_2_O catalysts substantially enhances their stability without significant cathode salt accumulation, highlighting the versatility and broad applicability of this salt suppression strategy.^[^ ^72^ ^]^

In the future, the urea community can benefit from two innovative CO_2_RR modules: i) hydrophobic‐to‐hydrophilic gradients that keep the outer GDE dry for CO_2_ penetration while wicking nitrate‐rich electrolyte into the catalyst layer, and ii) flow‐through GDEs with through‐plane macropores (<50 µm) that shorten the diffusion path of both gases and ions.

#### 3.4.3 Zero‑Gap MEAs; Collapsing Resistances and Synchronizing Proton Delivery

In the transition from GDE flow plates to a zero‐gap MEA, the distance between the gas‐fed cathode, the ion‐conducting membrane, and the anodic oxygen (or nitrate) evolution layer is reduced to less than 300 µm.^[^ ^73^ ^]^ It is essential to clarify that the “zero‐gap MEA” discussed in this paper refers to the core integrated component, which includes the cathode (typically a GDE), an ion‐conducting membrane, and the anode catalytic layer. This MEA serves as the primary site where electrochemical reactions occur. Conversely, the complete apparatus encompassing this MEA, along with bipolar plates (with flow channels), gaskets, seals, current collectors, and the external circulation system (pumps, tubing, storage tanks), is termed a “Flow Cell Electrolyzer”. The advantages and performance data described below are based on flow cell systems utilizing the zero‐gap MEA configuration. This geometry provides three significant advantages for the sixteen‐electron urea pathway: i) The ohmic drop is reduced to <0.5 Ω cm^2^, reducing the cell voltage by ≥100 mV at 200 mA cm^−^^2^ and keeping the specific energy below the 2 kWh kg_urea_^−^^1^ techno‐economic threshold. ii) Proton flux can be controlled with nanometre precision. Developers can place the acidification front where the *OCNO→ *NH‐CO protonation step occurs, while keeping the gas‐side boundary layer near neutral to prevent carbonate growth, by selecting the membrane type (Cation exchange membrane (CEM), anion exchange membrane (AEM), or bipolar) and water‐uptake number. iii) Gas and liquid residence times are separate. The GDE supplies CO_2_ on one side and circulates nitrate‐rich electrolyte through a thin catholyte channel on the other. The membrane prevents gas crossover while allowing selective ion migration, enabling the two feeds to be tuned independently, a feature not possible in thicker flow‐by designs. This dual‐pathway supply strategy is critical for synchronizing the delivery of gaseous CO_2_ and dissolved NO_3_^−^ reactants to their respective active sites at the catalyst layers. The typical flow field configuration (e.g., serpentine, parallel channels) is machined or stamped onto conductive plates (e.g., graphite, stainless steel, or coated titanium) that sandwich the MEA. These plates serve as current collectors and contain the channels that define the flow paths for the catholyte (liquid NO_3_^−^) and, in some designs, the anolyte. For the catholyte side supplying NO_3_^−^, the channel geometry (width, depth, path length) is optimized to ensure uniform distribution of nitrate across the catalyst surface under the required flow rates and pressure drops (typically <20 mbar), minimizing concentration gradients. Simultaneously, the CO_2_ gas is typically fed through a porous GDL backing the cathode catalyst layer, which may itself be integrated into or adjacent to a gas flow field on the opposite plate. The use of counter pressure helps suppress gas bubble formation within the liquid catholyte channel and maintains stable gas diffusion through the GDL.^[^ ^74^ ^]^

Anchor points that are currently at the forefront of technology are, for example, a nitrate‐to‐NH_3_ MEA with a nanostructured Ru‐Ni cathode and IrO_2_ anode that achieved 320 mA cm^−^^2^ at 95% FE (1.75 V cell) using a 50 µm PTFE‐reinforced CEM and 3 bar counter‐pressure on the gas side.^[^ ^74^ ^]^ Mass‐transport modelling indicates that identical ion‐migration coefficients will yield 150–200 cm^−^^2^. Early urea‐specific zero‐gap cells on oxide‐derived Cu reach 50 mA cm^−^^2^ at 24% FE.^[^ ^73^ ^]^ The performance gap is now traced to carbonate flooding rather than kinetic inactivity, highlighting the need for pH‐managed MEAs.

Knobs designed explicitly for the nitrate and CO_2_ pair. i) Water management. Nitrate reduction requires six protons per NO_3_^−^, while CO_2_ coupling requires an alkaline environment to stabilize *CO. The membrane must shuttle protons at centimeter‐scale current densities without flooding the GDE. Gradient hydration Nafion XL or Aquivion membranes (λ≈ 10–12) strike the balance, and their low swelling minimizes mechanical stress on the catalyst layer. ii) Bipolar membrane (BPM) modes. A thin BPM with forward bias generates protons at the catalyst‐membrane interface, reducing bulk electrolyte acidification and carbonate precipitation by 70% in 12 h of testing. Experimental evidence confirms that the zero‐gap BPM electrolyzer for urea synthesis fundamentally prevents carbonate precipitation through in situ CO_2_ bubble generation, which consumes bicarbonate ions. Using simulated flue gas (20% CO_2_ + 5% O_2_) at a full‐cell voltage of 2 V with 0.1 M KHCO_3_ + 500 ppm NO_3_^−^ electrolyte, the system demonstrated: stable pH (7.8–8.1) over 30 h of continuous operation without precipitation‐inducing fluctuations; absence of carbonate deposition confirmed by post‐operation SEM/TEM/XRD, showing unchanged morphology and structure of the Cu_95_Ru_5_ catalyst; enhanced performance with FE_urea_ reaching 58% (simulated flue gas) and 62% (pure CO_2_) at a stable current density of 20 mA cm^−^^2^. This significantly outperforms conventional H‐cell systems (19.52% FE_urea_).^[^ ^73^ ^]^

Pulsed reverse bias can remove carbonate by briefly making the junction alkaline. This mode maintained stable operation for 236 h, while constant voltage mode (without pulsing) failed within 10 h due to salt blockage. The current density fully recovers after each regenerative phase, confirming the dissolution of salt deposits. Model analysis validated the migration of carbonate ions from the cathode zone via electromigration during regeneration.^[^ ^75^ ^]^ iii) Integrated shims and flow fields. Stainless‐steel or PEEK shims with 100–300 µm serpentine channels distribute nitrate uniformly, maintain a pressure drop of less than 20 mbar, and act as mechanical buffers to prevent membrane wrinkling, especially for plasma‐derived feeds with fluctuating flow rates. The serpentine flow field drives transverse CO_2_ diffusion within the GDL by generating a significantly higher pressure drop (936% greater than parallel flow fields). This effectively restricts the catalyst area deactivated by KHCO_3_ deposition to only 3% (compared to 8% in parallel flow fields). Evaluation via Electric Double‐Layer Capacitance (EDLC), which reflects GDL wetting (indicative of reaction flooding tendency and positively correlated with salt deposition), revealed that the serpentine channel's capacitance reached only 0.98 mF cm^−^^2^ after 30 minutes. In contrast, parallel/cross‐flow channels exhibited a capacitance of 2.8 mF cm^−^^2^, a threefold increase, approaching that of catalyst‐free carbon GDL (3.7 mF cm^−^^2^). This indicates severe flooding (accelerating salt deposition). The serpentine flow field maintained a CO FE >60% for 1 h at 300 mA cm^−^^2^ (vs a rapid decline to 19.7% in parallel flow fields), demonstrating over a threefold improvement in clogging resistance. This design represents the most effective flow field configuration for mitigating performance decay caused by carbonate deposition.^[^ ^76^ ^]^

#### 3.4.4 Integration with Plasma Output Streams: Matching a Fluctuating NO_x_ Feed to Steady Electrolysis

Non‐thermal discharges exhale a warm, humid mixture that contains residual O_2_ and 0.5–5 vol% NO and NO_2_. Before this stream can serve the cathode, it must i) cool to ambient temperature, ii) convert NO to the more soluble NO_2_, iii) quantitatively dissolve NO_x_ as NO_3_^−^/NO_2_^−^, and iv) remove the final traces of O_2_ that would oxidize *CO intermediates. The most energy‐efficient solution reported so far combines a micro‐bubble Venturi absorber (ΔP < 20 kPa) with an upstream Mn‐Cu spinel oxidation bed, resulting in >95 % NO→NO_3_^−^ conversion and <0.5 ppm O_2_ slip at throughputs of 1–10 NL min^−^^1^. The absorber effluent provides a controllable 0.1–1 M nitrate feed that is blended with recirculated catholyte to mitigate minute‐scale plasma fluctuations (±5%). At the same time, inline pH/NO_3_^−^ sensors adjust either the plasma duty cycle or electrolyzer current to maintain the NO_3_^−^∶CO_2_ ratio and ionic strength within the catalyst's optimum window.^[^ ^7a^ ^]^ Closed‐loop integration, already used in pilot ammonia stacks, is crucial for µ‐modular urea units co‐located with wind or solar farms, where power availability and plasma output fluctuate on a sub‐hour scale.

#### 3.4.5 Urea Collection and Enrichment: Mitigating Downstream Barriers

For electrolytically synthesized urea to serve as a drop‐in liquid fertilizer, downstream designs must address concentration, decomposition, and compatibility with field equipment while leveraging electrolyzer innovations.

The key design considerations for downstream collection or enrichment units for urea lie in: i) In‐line concentration to avoid thermal degradation. Urea decomposes into NH_3_ and HNCO (isocyanic acid, reducing nitrogen content) at temperatures >60 °C.^[^ ^77^ ^]^ Conventional thermal evaporation is therefore unsuitable. Waste heat (<50 °C) from the plasma/electrolyzer can be utilized to drive an integrated low‐temperature membrane distillation (MD) process, concentrating urea to 20–30 wt% (a field‐applicable concentration).^[^ ^78^ ^]^ In coastal regions, a forward osmosis (FO) approach can alternatively be employed: using seawater as the draw solution to achieve 85% water removal at 35 °C.^[^ ^79^ ^]^ ii) Carbonate/nitrate removal for product stability. Residual CO_3_^2^^−^ (from CO_2_RR) and NO_3_^−^ (from incomplete conversion) in the product stream form precipitates that can clog nozzles and alter soil pH. Bipolar membrane electrodialysis (EDBM) is implemented in the catholyte loop to separate neutral urea from ionic species: CO_3_^2^^−^ → CO_2_(g) + OH^−^, NO_3_^−^ → HNO_3_ (recovered for pH control). This achieves >95% ion removal at 30% lower energy versus thermal evaporation (representing a 1.5 times higher removal rate compared to thermal methods).^[^ ^80^ ^]^ iii) Cation removal for product purity. Typical K⁺ concentrations in urea‐enriched streams (0.1–1 M) far exceed acceptable limits for fertigation and industrial feedstocks. Elevated K⁺ can induce soil salinity stress, accelerate corrosion of storage and injection equipment, and complicate downstream crystallization. To reduce K⁺ to <10 mM levels, several separation technologies can be coupled post‐electrolysis: Cation‐exchange electrodialysis (CED) utilizes stacks of cation‐exchange membranes to selectively migrate K⁺ ions, achieving over 90% removal in a single pass while producing a concentrated potassium fertilizer stream as a byproduct.^[^ ^81^ ^]^ Strong‐acid cation‐exchange resins, such as sulfonated polystyrene beads, leverage their high exchange capacity of up to 2 meq·g^−^^1^; continuous fixed‐bed adsorption with these resins can subsequently polish residual potassium ions down to levels below 1 ppm.^[^ ^82^ ^]^ The membrane separation domain offers two complementary approaches: RO achieves >99% rejection of monovalent ions at an energy consumption of 1–2 kWh·m^−^^3^, though careful control of urea permeation loss is required.^[^ ^83^ ^]^ Capacitive deionization (CDI), on the other hand, relies on the electrosorption characteristics of carbon electrodes under low voltage (<1.2 V), enabling modular low‐temperature operation that removes 70–80% of cations per adsorption cycle.^[^ ^84^ ^]^ In practical applications, technology integration should be guided by the required purification depth, energy costs, and the need for product recovery. Integrating these cation‐removal units with existing bipolar ED and membrane‐distillation steps yields a final urea product (25–30 wt%) with K⁺ concentrations <10 mM, fully compliant with UAN specifications and minimizing downstream corrosion risks. iv) Compatibility with existing infrastructure. The collected liquid urea (25–30 wt%) matches commercial products (e.g., UAN‐32). Residual K⁺ ions in the electrolyte serve as a potassium fertilizer supplement. Furthermore, adding 0.1–0.3 wt% phosphoric acid (H_3_PO_4_) at the electrolyzer outlet inhibits hydrolysis (maintaining pH 3–4) while providing value as a phosphorus fertilizer. Critically, the low‐temperature processing during collection prevents biuret formation (<0.5%, compliant with China National Standard GB/T 15063‐2009), ensuring product safety and regulatory compliance.^[^ ^85^ ^]^ Integrating urea recovery as a value‐added extension of the electrolyzer design, directly enabling scalable, solar‐powered liquid fertilizer production.

#### 3.4.6 Strategies and Recommendations for Urea Electrosynthesis Electrolyzers

To address the unique complexities of the 16‐electron, 18‐proton urea electrosynthesis pathway, targeted design strategies must be developed based on insights from simpler electrochemical technologies. Hydrophobic GDEs with microporous layers that exhibit high contact angles (>115°) are necessary to minimize electrolyte flooding and maintain stable triple‐phase boundaries over extended periods of operation. Using a pressurized cathode (2–3 bar) improves CO_2_ solubility and reduces carbonate precipitation. This addresses the increased carbonate formation caused by the dual‐feed scenario in urea electrolysis.

Furthermore, using advanced elastic ion‐exchange binders, such as perfluoro‐alkylated styrene ionomers, ensures strong adhesion of the catalyst layer, thereby preserving catalytic integrity even in turbulent electrolyte flows. The transition to zero‐gap or minimal‐gap MEAs is critical for significantly reducing ohmic losses and precisely controlling proton delivery, which directly facilitates the protonation of key intermediates such as *OCNO. Inline absorption and separation units, such as microbubble reactors or Venturi systems, are recommended to convert plasma‐generated NO_x_ gases into stable nitrate solutions, ensuring consistent feedstock concentrations. Using sequential reaction staging in single electrolyser systems enables the direct coupling of plasma nitrate reduction and subsequent CO_2_ coupling reactions, resulting in increased system efficiency and energy utilization.

#### 3.4.7 Future Research Directions and Opportunities

Concentrated research efforts are necessary in several key domains to close the critical gap between commercially viable electrolyzer technologies and promising laboratory‐scale demonstrations. The primary goal is to create fully integrated plasma‐electrolyzer modular systems that match plasma‐generated nitrate feed concentrations to electrolyzer demand, eliminating the need for costly intermediate storage. Advanced dynamic modelling and control strategies will be required to synchronize fluctuating plasma output streams with steady‐state electrolyzer consumption precisely.

Further advancements in electrolyte formulations and membrane technologies are required to manage localized pH gradients, stabilize sensitive reaction intermediates, and reduce carbonate accumulation, which impairs long‐term performance.^[^ ^65^, ^86^ ^]^ Furthermore, advancements in operando diagnostics and in situ analytical techniques, such as Raman spectroscopy, infrared spectroscopy, and mass spectrometry, will allow for real‐time monitoring of catalyst surface reactions, intermediate speciation, and byproduct formation.^[^ ^87^ ^]^ Such analytical capabilities will provide valuable feedback for rapid system optimization and catalyst refinement. Electrochemical urea synthesis can overcome current technical and operational limitations by leveraging knowledge from related electrochemical technologies, especially advanced CO_2_ and nitrate reduction reactors. Integrating detailed design principles, material innovations, and system‐level engineering advancements will drive the commercialization of robust, efficient, and scalable electrolyzer technologies for long‐term green urea production.

## 4 Techno‐Economic Price Projection and Life‐Cycle Analysis

Through an integrated techno‐economic analysis and life‐cycle assessment, this section assesses the economic and environmental advantages. Our techno‐economic analysis uses realistic experimental metrics for i) non‐thermal plasma oxidation of air to NO_x_ and ii) electrocatalytic co‐reduction of nitrate and CO_2_ to urea. Full model details are available in the Supporting Information.

### 4.1 Techno‐Economic Analysis

Key benchmark assumptions: Our plasma step uses a conservative specific energy of 2.0 MJ mol^−1^ NO_x_. This value reflects today's cutting‐edge non‐thermal systems, considering gas‐to‐liquid absorption losses. Historical data show steady progress: early Birkeland‐Eyde arcs consumed ≈3 MJ mol^−^^1^,^[^ ^11^ ^]^ while gliding arc and spark reactors have fallen to 2.9–1.9 MJ mol^−^^1^.^[^ ^27^ ^]^ Recent microwave discharges report 1.17–1.78 MJ mol^−^^1^.^[^ ^23^, ^24^ ^]^ At the observed learning rate (≈6% per year over the last decade), achieving ≈1 MJ mol^−^^1^ within five years is feasible. For the electrochemical stage, we chose 50% FE and 60% N‐selectivity to urea, which are within the current ranges of 30–80% FE and 40–90% selectivity. The current global average grid electricity price is US$ 0.065 kW h^−^^1^. The impact of lower renewable tariffs will be analyzed further.

**Figure 10:**
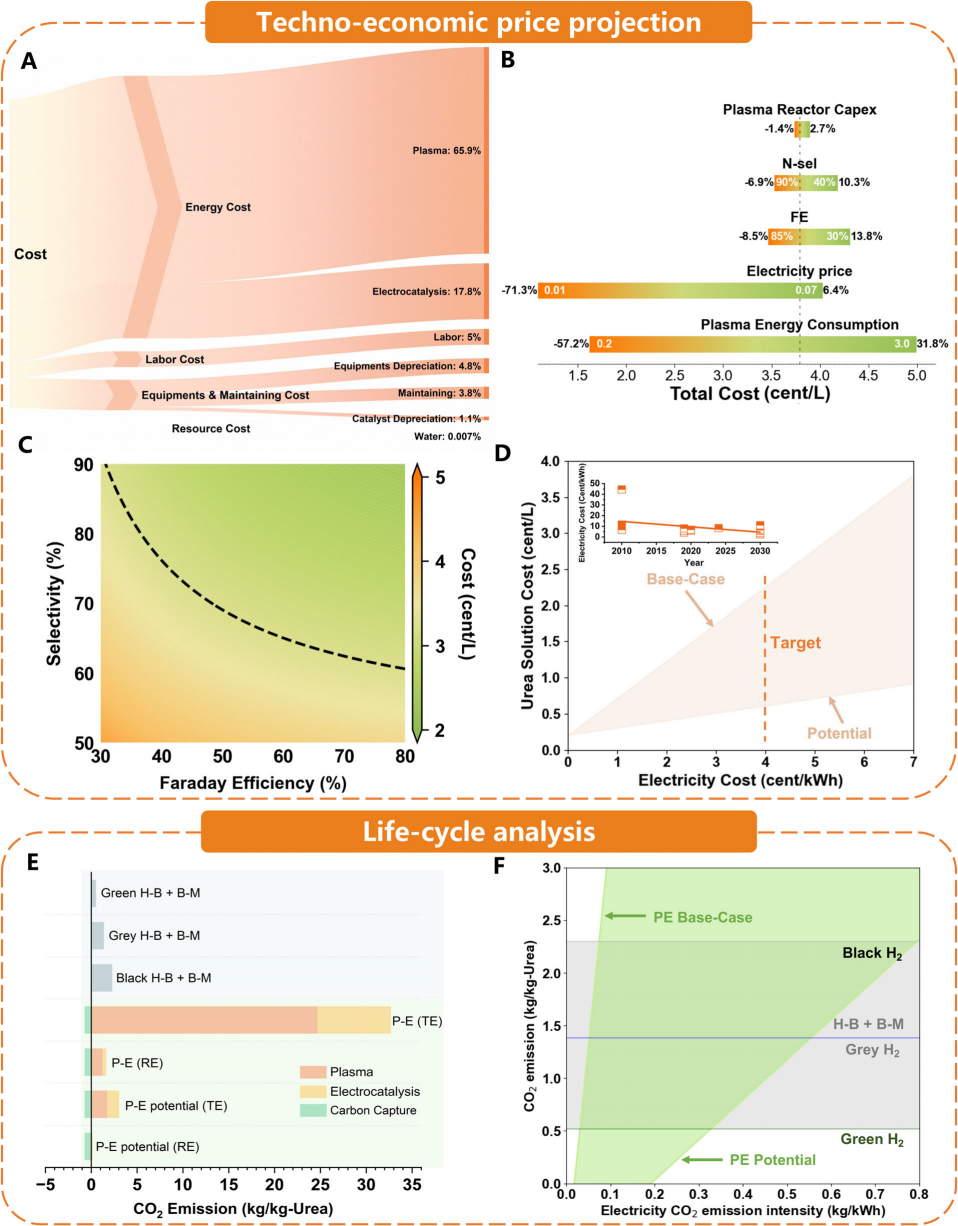
Cost & CO_2_‐intensity heat map. A) Cost ratios of the entire production process of liquid urea fertilizer for a given electricity price of 0.065 $ kWh^−^^1^, B) Sensitivity analysis of cost, highlighting key cost drivers and parameter sensitivities, C) Two‐point sensitivity analysis: the cost of synthesising liquid urea as a function of Faraday efficiency and N‐selectivity for a two‐step strategy. The black dashed line represents the minimum market price level for liquid urea, i.e., the cost of generating 3.21 cent L^−^^1^ liquid urea, D) Sensitivity analysis of electricity prices, E) Equivalent CO_2_ emissions (CO_2e_) for PE green urea production versus conventional urea production, F) Carbon emissions under different electricity carbon intensity conditions.

Cost structure: The model categorizes expenses as energy, capital + maintenance, labor, and feedstock (**Figure** **10**A). According to the baseline assumptions, plasma NO_x_ accounts for ≈44%, and electrocatalysis accounts for ≈18% of the total cost. Capital depreciation and O&M account for less than 10%, while raw material costs are minimal (0.007%), as the only consumables are air, water, and captured CO_2_. These figures highlight that the economic case for plasma‐electrolysis urea relies heavily on continued reductions in plasma‐energy demand and improvements in electrolyzer efficiency.

#### 4.1.1 Sensitivity Analysis of Cost: The Economic Effects of Plasma Reactor Operation

The plasma oxidation step is the most energy‐intensive component in our two‐step urea synthesis process, consuming nearly half of the total electrical input and driving roughly 44% of the levelized production cost under baseline conditions. Explicitly isolating and quantifying the plasma reactor's operating expenses is therefore essential to prioritize cost‐reduction efforts, inform technology road‐mapping and mapping plasma operating costs against historical and projected energy efficiencies clarifies the economic payback of continued reactor development (e.g., advanced vibrational excitation, optimized electrode design) and supports strategic investment in next‐generation discharge techniques.

As shown in Figure 10B, under our baseline assumptions (2.0 MJ NO_x_^−^^1^ specific energy; grid electricity at US$ 0.065 kWh^−^^1^), the plasma reactor consumes on the order of 127 MWh of electricity per day—equating to an operating expense of approximately US$ 8240 day^−^^1^. Spread over the daily production of 342 000 L of 1.2 wt % urea solution, this corresponds to 2.4 ¢ L^−^^1^, making the plasma step the single largest cost driver (44% of total) in the levelized cost profile.

Sensitivity analysis underscores the critical leverage held by plasma energy efficiency as below:

Mid‐range improvements: Reducing specific energy from 2.0 to 1.0 MJ NO_x_^−^^1^ cuts plasma operating costs by nearly 50%, saving ≈1.2 ¢ L^−^^1^.

Theoretical minimum: Approaching the vibrationally optimized limit of 0.2 MJ NO_x_^−^^1^ yields cost reductions exceeding 60% (over 2.2 ¢ L^−^^1^), dramatically lowering the levelized cost of urea.

Efficiency regression: Conversely, a return to older arc technologies at 3.0 MJ NO_x_^−^^1^ would raise operating expenses by over 1.2 ¢ L^−^^1^, quickly eroding commercial viability.

These results demonstrate that reducing plasma‐specific energy is the critical lever for driving the levelized cost of green urea production toward competitive benchmarks. Continuous optimization of reactor energy efficiency, alongside strategic electricity procurement and demand‐response operation, will therefore be the primary focus for achieving economically viable plasma‐electrocatalytic urea synthesis.

In addition to operating expenses, the capital investment for the plasma reactor can also affect the levelized cost of urea, albeit modestly. Our baseline assumes a combined plasma‐reactor and equipment capex of $1.30 M, with straight‐line depreciation over 10 years. This corresponds to about $356 day^−1^ in fixed capital charge, or roughly 0.10 ¢ L^−1^ when spread over 342 000 L day^−^^1^ production. Even large swings in reactor capex yield only small cost impacts: for example, halving or doubling the investment (to about $0.65 M or $2.6 M) would change the levelized cost by only ≈0.05 ¢ L^−^^1^ downward or upward, respectively (i.e., from ≈0.05 to ≈0.15 ¢ L^−^^1^). For context, Previous study suggests that plasma reactors can cost on the order of ≈$1300 per kW of power, which for a multi‐megawatt system would imply several million dollars of equipment.^[^ ^88^ ^]^ Thus, even if reactor capex were as high as $2–3 M, the depreciation charge adds only a few hundredths of a cent per liter, much smaller than the ≈1–2 ¢ L^−^^1^ effects of energy or performance (FE, N‐selectivity) improvements. We therefore include “Plasma Reactor Capex” as a sensitivity category. The result shows that ±50% changes in the $1.3 M reactor capex alter the cost by only a few hundredths of a cent, compared to tenths of cents for the other drivers. This confirms that plasma‐reactor capex is a minor cost lever relative to energy consumption and conversion efficiency.

#### 4.1.2 Two‐Factor Sensitivity: Where Catalytic Progress Pays the Significant Dividend

A two‐factor sensitivity map (Figure 10C) illustrates the levelized cost of urea within today's realistic operating envelope, with a 30–80% FE range and nitrogen selectivity of 50–90.^[^ ^9^, ^89^ ^]^ Over the past decade, FE has increased from less than 10% in first‐generation batch cells (2015) to 50–60% on relay‐site GDEs and vacancy‐patched oxides (2024), with a compound annual growth rate of ≈11%.^[^ ^5^, ^43^ ^]^ Nitrogen selectivity has tripled from ≈20 % to ≈60 % over the same period, resulting in a ≈13% year^−^^1^ improvement driven by dual‐site engineering and dynamic bias protocols.^[^ ^10^ ^]^

When we apply historical gains to our cost model, a 10‐point increase in N‐selectivity reduces the levelized cost by ≈0.6 ¢ L^−^^1^, which is twice the benefit of a comparable jump in FE. Higher selectivity reduces nitrate electron waste as NH_3_, resulting in a decrease in the plasma power bill, a major operating expense. When the catalyst achieves ≥80% N‐selectivity, the production cost falls below the current market floor of 3.21 ¢ L^−^^1^, even if FE remains at a moderate 55%.

By 2028, laboratory catalysts are expected to achieve 80% selectivity if research prioritizes suppressing NH_3_ and CO side paths through strategies such as relay metal pairs, vacancy‐tuned protonation barriers, and work‐function‐matched heterointerfaces, according to a projected 13% compound annual growth rate. In short, what electrons build is now more valuable than how many arrive. Perfecting the OCNO into a urea manifold unlocks economic viability faster than pursuing incremental gains in charge efficiency.

#### 4.1.3 Learning‐Curve Outlook and the Impact of Declining Renewable Electricity Prices

RE costs have declined significantly over the past decade, with the levelized costs of electricity (LCOE) from solar photovoltaic and onshore wind technologies decreasing from approximately US$0.13 kWh^−^^1^ in 2013 to around US$0.04 kWh^−^^1^ in 2023.^[^ ^90^ ^]^ Policy‐driven roadmaps aim for even lower electricity prices, with a target of around US$0.02 kWh^−1^ in 2030.^[^ ^91^ ^]^ Regions with high renewable energy penetration, such as Spain, Chile, and Western Australia, already have negative wholesale electricity prices during peak renewable generation periods.^[^ ^92^ ^]^ Against this backdrop, we created the “PE Potential” scenario, which envisions significant technological advancements possible within a similar timeframe. This scenario features optimized plasma reactor efficiency, approaching the theoretical minimum of 0.2 MJ mol^−^^1^ NO_x_ through advanced vibrational activation techniques. It also achieves elevated electrocatalytic performance, with a FE of 85% and nitrogen selectivity. Additionally, RE is priced at around US$0.01 kWh^−^^1^, representing the midday solar surplus in future grids.

Figure 10D shows the significant economic benefits of converging technological and market trends. At current average electricity tariffs (≈US$0.065 kWh^−^^1^), the production costs of plasma‐electrocatalytic urea synthesis are ≈4 cents per liter. However, under the “PE Potential” assumptions, the costs fall dramatically below 1 cent per liter, representing a 70% reduction and clearly demonstrating the economic viability that can be achieved through continued technological advancements and renewable energy integration.

#### 4.1.4 Time‐of‐Use Optimization and Demand‐Flexible Operation

The changing dynamics of electricity markets favor demand‐flexible operations, particularly in areas with high renewable energy integration. In Germany, the frequency of negative day‐ahead electricity prices has increased dramatically, with 301 h recorded in 2023, up from 139 h in 2022. Similarly, in California, the integration of significant solar capacity resulted in periods of negative wholesale electricity prices. For example, in Southern California, wholesale prices fell for nearly 20% of all hours in 2023. Similar trends have emerged in Australia's National Electricity Market. In the third quarter of 2023, negative wholesale electricity prices occurred in 19% of all dispatch intervals, the highest frequency ever recorded. This increase is due to higher outputs from variable renewable energy sources and lower operational demand, particularly during the midday hours.^[^ ^93^ ^]^ These market conditions provide opportunities for energy‐intensive processes, such as plasma‐electrocatalytic urea synthesis, to improve efficiency. Plasma systems’ inherent flexibility allows for quick modulation of power input, allowing operations to coincide with periods of low or negative electricity prices. Strategically scheduling production during these times can save energy costs without requiring additional capital investment. Thus, the most immediate practical path to commercial competitiveness is presented by combining real‐time electricity pricing signals with a modest nitrate buffering capacity, as discussed in this perspective, which leverages ongoing technological advances.

### 4.2 Life‐Cycle Assessment

#### 4.2.1 Environmental Impact

Our life‐cycle assessment compares the carbon footprint (CO_2_‐equivalent emissions, CO_2_e) of plasma‐electrocatalytic urea production with conventional Haber–Bosch processes from an environmental perspective (Figure 10E). The traditional urea synthesis methods, which rely on hydrogen derived from fossil fuels, produce significant CO_2_e emissions. These emissions range from ≈2.3 kg CO_2_e kg_urea_^−^^1^ (coal‐derived black hydrogen) to 0.33 kg CO_2_e kg_urea_^−^^1^ (renewable‐driven green hydrogen).^[^ ^94^ ^]^ In contrast, plasma‐electrocatalytic urea synthesis, which is powered by RE, has the potential to significantly reduce emissions to ≈1.2 kg CO_2_e kg_urea_^−^^1^ under current baseline scenarios. In optimized “potential” scenarios, emissions can be reduced even further to 0.3 kg CO_2_e kg_urea_^−^^1^, with CO_2_e emissions dropping to 2.8 kg CO_2_e kg_urea_^−^^1^ even under thermal electricity (TE) conditions.^[^ ^95^ ^]^ This substantial reduction highlights the significant environmental benefits of renewable‐driven plasma‐electrocatalytic urea synthesis compared to conventional fossil‐based methods.

#### 4.2.2 Carbon Intensity Considerations

Our sensitivity analysis (Figure 10F) further illustrates the fundamental impact of the carbon intensity of electricity on the environmental competitiveness of plasma‐electrocatalytic urea synthesis. Currently, the most significant environmental benefits are achieved by utilizing ultra‐low carbon‐intensity electricity (<0.05 kg CO_2_ kWh^−^^1^), which is typical of hydropower. Nevertheless, plasma‐electrocatalytic synthesis remains environmentally advantageous across a broader spectrum of electricity carbon intensities under the optimized technological conditions in the “PE Potential” scenario. This highlights the crucial importance of ongoing enhancements in electrocatalytic selectivity and plasma efficiency to expand the environmental benefits further.

To quantify the effect of regional electricity carbon intensity, we consider representative high‐emission grids. The carbon intensity of electricity generation varies widely by country and state. For example, in India (77–80% fossil generation) the power sector emitted ≈713  gCO_2_ kWh^−^^1^ in 2021.^[^ ^96^ ^]^ China's coal‐heavy grid averaged ≈537  gCO_2_ kWh^−^^1^ in 2020.^[^ ^97^ ^]^ In the U.S., the 2023 national average was ≈0.37  kgCO_2_ kWh^−^^1^, but states like Wyoming, Kentucky, and Indiana (with large coal shares) had much higher intensities (≈846, 789, and 679  gCO_2_ kWh^−^^1^, respectively).^[^ ^98^ ^]^ These values contrast with grids dominated by renewables/hydro, which can be <100  gCO_2_ kWh^−^^1^.

Using these intensities, we calculate plasma‐urea CO_2_ emissions as *E·I*, where *E* is the electricity consumption per kg urea and *I* the grid intensity. For illustration, assume *E*≈15 kWh kg^−^^1^. Under a coal‐intensive grid (I≈0.8 kgCO_2_ kWh^−^^1^), this implies ≈12 kgCO_2_ per kg urea (12 t CO_2_ t_urea_^−^^1^). At I = 0.5 kg kWh^−^^1^ (e.g., moderate fossil mix), ≈7.5 kg kg^−^^1^; at I = 0.3 kg kWh^−^^1^ (typical of cleaner regional mixes), ≈4.5 kg kg^−^^1^; at I = 0.1 kg kWh^−^^1^ (very clean), ≈1.5 kg kg^−^^1^. In the best case of near‐zero‐carbon power (e.g., 50 g kWh^−^^1^), emissions drop below 1 kg kg^−^^1^; with true renewables or nuclear (I→0), the process approaches zero CO_2_. Thus, the range of plasma‐urea emissions spans roughly 0–12  kgCO_2_ kg^−^^1^ depending on location. These estimates are substantially higher than the ≈0.9–1.1 kg kg^−^^1^ emitted by current fossil‐based urea synthesis.

These results imply that plasma‐electrocatalytic urea can only deliver net climate benefits in low‐carbon grids. In coal‐dependent markets (e.g., parts of India, China or U.S. coal states), the life‐cycle emissions of urea via plasma would greatly exceed those of conventional production. Only where cheap renewable or nuclear power is available (as recent LCOE trends suggest in many regions can plasma‐urea approach carbon neutrality.^[^ ^99^ ^]^ Global deployment of this technology must be paired with grid decarbonization: in effect, the achievable CO_2_ intensity of urea is tied directly to that of the local electricity supply. These quantitative scenarios underscore that the environmental advantage of plasma‐based urea is region‐specific and contingent on low‐carbon electricity (≤≈50  gCO_2_ kWh^−^^1^).^[^ ^96^, ^98^ ^]^

## 5 Research Roadmap and Open Questions for the Community

As outlined in this perspective, plasma‐enabled electrocatalytic urea synthesis represents a promising pathway to a sustainable and decentralized fertilizer industry. To help the research community, industry stakeholders, and policymakers achieve commercial viability and widespread adoption, we outline critical research directions organized through a diagnostic lens: identifying current bottlenecks, modular approaches to solutions, leveraging cross‐disciplinary innovations, and defining targeted research directions to achieve techno‐economic and environmental parity with conventional methods.

Reducing energy consumption in the plasma‐driven NO_x_ synthesis step is the main challenge. Despite recent advances, current plasma reactors operate at 1–3 MJ mol^−^^1^, which is still far from the theoretically achievable 0.2 MJ mol^−^^1^ limit.^[^ ^11^ ^]^ Recent breakthroughs in microwave and gliding‐arc systems have demonstrated the need for research to focus on optimized reactor geometries, nanosecond pulse modulation, resonant microwave coupling, and enhanced vibrational pumping.^[^ ^12^, ^13^, ^26^ ^]^ Cross‐disciplinary insights from plasma catalysis and atmospheric chemistry should be thoroughly investigated to refine electron energy distributions, reduce recombination losses, and create energy‐efficient absorber designs.

The electrocatalytic step also presents significant challenges. Current catalyst designs achieve moderate FEs (50–60%) and selectivities (60–70%), which are insufficient for immediate commercial applications.^[^ ^1^, ^2^ ^]^ Targeted research must prioritize dual‐site and relay‐site catalyst architectures that stabilize critical intermediates (*OCNO, *NH‐CO) while suppressing competing pathways to ammonia or formate. Leveraging established strategies from the fields of CO_2_ electroreduction and nitrate reduction, such as facet engineering, defect‐rich oxide materials, electronic gating at interfaces, and dynamic voltage biasing, provides immediate and impactful opportunities. Future research should concentrate on the operando identification and control of rate‐determining steps, utilizing advanced spectroscopic and computational techniques to fine‐tune catalyst structures and electronic properties.

From a systems engineering perspective, robust electrolyzer designs tailored for dual‐feed scenarios require immediate development. Hydrophobic GDEs, advanced microporous layers, pressurized cathode operation, and zero‐gap MEAs have been successfully demonstrated in flow batteries and CO_2_ electrolyzers.^[^ ^60^, ^61^, ^68^, ^100^ ^]^ urea synthesis presents unique challenges, including severe carbonate fouling and precise nitrate and CO_2_ mass flow synchronization. Integrating inline, real‐time analytical protocols (for example, dual‐probe colorimetric/HRMS detection and isotope‐tracing methodologies) will ensure accurate, interference‐free product quantification, laying the groundwork for scale‐up.

Techno‐economic modeling highlights key milestones, including achieving plasma efficiencies below 1 MJ mol^−^^1^ NO_x_, exceeding 70% nitrogen selectivity, and using RE priced below US $35 MW h^−^^1^. Meeting these targets necessitates an integrated research strategy that combines plasma reactor development, catalyst durability optimization, electrolyzer mass‐transport engineering, and dynamic control systems designed for intermittent renewable energy sources.

Policymakers and funding agencies should be aware of the significant societal and environmental implications of this research direction. Structured policy support, clear regulatory frameworks for green fertilizers, and strategic public‐private partnerships are critical for accelerating research translation, incentivizing early‐stage technology adoption by industry, and increasing community acceptance. Public awareness campaigns emphasizing the environmental and sustainability benefits will also be critical in generating the societal momentum required to support this transformational shift in fertilizer production.

The roadmap we propose is more than just a research agenda; it represents a collaborative path to sustainability that connects scientific innovation, industrial practice, and informed policymaking. With targeted investment, sustained interdisciplinary effort, and clear technological benchmarks, plasma‐enabled electrocatalytic urea synthesis has the potential to redefine the fertilizer industry, firmly establishing it within a decarbonized and electrified global economy [Supplementary-material adma70244-supitem-0001].

## Conflict of Interest

The authors declare no conflict of interest.

## Supporting information

Supporting Information
