# Supplementary material for: A Roadmap for Plasma‐Enabled Electrocatalysis in Urea Production
Source: Adv Mater. 2025 Aug 13;37(43):e09784. doi: 10.1002/adma.202509784 (PMC12574632; doi:10.1002/adma.202509784)
Supplement: Supplementary file 1 — Supporting Information [file ADMA-37-e09784-s001.docx]

Supporting Information

A roadmap for plasma-enabled electrocatalysis in urea production

Jingwen Huang, Zhongping Qu, Renwu Zhou*, Jing Sun, Rusen Zhou, Dorna Esrafilzadeh and Ali R. Jalili*

**Cost Estimation**

**1. Production Capacity and Urea Yield**

The plant is designed to produce one billion liters per year of a 1.2 wt % urea solution. With a capacity factor of 0.80, the facility actually turns out eight hundred million liters of product each year. Spread over 365 days, this corresponds to roughly 342 000 L of urea solution every day, which at 1.2 % concentration delivers about 4.1 tonnes of pure urea per day (equivalent to approximately 68 000 mol of urea). In order to satisfy the nitrogen requirement for that yield—recognizing each mole of urea contains two moles of nitrogen and assuming 60 % selectivity to nitrate—some 228 000 mol of nitrogen (as NO₃⁻) must be supplied daily.

**2. Raw-Material Costs**

Since the only feedstocks for this process are air and water, raw‐material costs are minimal. Air is freely available, and high‐purity water consumption is the only consumable expense. At an approximate usage rate of 343 t/day and a typical agricultural water tariff of 0.0015 $/kg, the daily water cost amounts to just 0.51 $.

**3. Energy Consumption and Expenditure**

- **Plasma-Driven Oxidation**

Generation of reactive nitrate species by plasma requires about 2 MJ of electrical energy per mole of NO₃⁻. To meet the daily demand of roughly 228 000 mol, the plasma unit draws on the order of 127 000 kWh each day. At an industrial electricity rate of 0.065 $/kWh, this translates into a daily charge of approximately 8240 $.

- **Electrocatalytic Urea Formation**

Converting nitrate into urea electrochemically involves transferring 16 electrons for each mole of urea. Accounting for a 50 % Faradaic efficiency, the cell consumes around 41 000 kWh per day at an operating voltage near 0.7 V. This portion of the process thus incurs about 2680 $ in electricity costs each day.

Altogether, electrical energy accounts for about 10920 $ of the daily operating expense.

**4. Capital Investment and Maintenance**

The combined capital cost for the plasma reactor, high-voltage pulse supply, electrochemical cell, and auxiliary equipment is estimated at 1.30 million $. Assuming a ten-year design life and straight-line depreciation, this amounts to approximately 356 $/day. Routine maintenance is budgeted at 8 % of the initial capital each year, adding roughly 285 $/day. Thus, equipment ownership and upkeep together contribute about 641 $/day.

**5. Labor**

Labor costs are assumed to be 5 % of all other expenditures (energy, water, equipment), yielding a daily payroll expense of approximately 609 $.

**A direct comparison to Haber-Bosch/Bosch-Meiser routes**

| Route | Energy Consumption | CAPEX | OPEX | Operational Flexibility |
| --- | --- | --- | --- | --- |
| Haber–Bosch + Bosch–Meiser (conventional) | ≈ 0.3 GJ/t^[1]^ | ~ 1 00 $/t | ~150 $/t | Low (large baseload) |
| Plasma‐Electrocatalysis (Baseline) | ≈ 1.77 GJ/t | ~ 1.04$/t | 34.6 $/t | High (modular, fast ramp) |
| Plasma‐Electrocatalysis (PE Potential) | ≈ 0.39 GJ/t | ~ 1.04$/t | 1.94 $/t | High (modular, fast ramp) |

**Table S1.** A direct comparison to Haber-Bosch/Bosch-Meiser routes.

**Research Status on Plasma Driven Air to NO_x_**

| Cathode/  Catalyst | NOx Concentration  (ppm) | Energy Cost  (MJ/mol) | Ref. |
| --- | --- | --- | --- |
| Spark(Abdelaziz,2024) | 125.57 | 51.97 | ^[2]^ |
| Spark(Abdelaziz,2024) | 53.2 | 72.1 | ^[2]^ |
| Spark(Abdelaziz,2024) | 22.34 | 50.1 | ^[2]^ |
| Spark(Zhang，2022) | 21.04 | 51.27 | ^[3]^ |
| Spark(Abdelaziz,2024) | 73.5 | 75.6 | ^[4]^ |
| RGA (Jardali, 2021) | 41.6 | 55.8 | ^[5]^ |
| RGA(Denra,2024) | 26.74（-0.6V） | 79.01 | ^[6]^ |
| GA(Vervloessem,2020) | 25.6 | 46.1 | ^[7]^ |
| GA(Vervloessem,2020) | 1.64 | 70.1 | ^[7]^ |
| GA(Vervloessem,2020) | 298.67 | 31.71 | ^[7]^ |
| RA(Muzammil, 2021) | 15.16 | 60.6 | ^[8]^ |
| RGA(Majeed,2024) | 25.34 | 34.1 | ^[9]^ |
| GA(S-Li, 2023) | 22.9 | 88.5 | ^[10]^ |
| RGA(Tsonev,2023) | 30.6 | 34.5 | ^[11]^ |
| RGA (Alphen ,2022) | 23.3 | 57.02 | ^[12]^ |
| GA(Manaigo,2025) | 135.6 | 90.0 | ^[13]^ |
| GA(Angineni,2025) | 58.09 | 73.69 | ^[14]^ |
| MW (Kelly,2021) | 61.6 | 75.6 | ^[15]^ |
| MW(Li,2025) | 33.30 | 54.1 | ^[16]^ |
| MW(Wu,2025) | 103.1 | 11.9 | ^[17]^ |
| DBD((Abdelaziz,2023) | 27 | 66.5 | ^[18]^ |
| DBD(Li.2023) | 36.92 | 54.3 | ^[19]^ |

**Table S2.** Research Status on Plasma Driven Air to NO_x_.

**Research Status on Electrocatalytic Synthesis of Urea from NO₃⁻ and CO₂**

| Cathode/  Catalyst | FE (%) | Current Density  (mA/cm^2^) | Applied potential  (V vs. RHE) | Stability  (h) | Ref. |
| --- | --- | --- | --- | --- | --- |
| 6Å-Cu | 51.97 | 115.25 | -0.41 | 50 | ^[20]^ |
| CuPd_1_Rh_1_-DAA | 72.1 | 20 | -0.5 | 100 | ^[21]^ |
| Ru_1_/Co | 50.1 | 10 | -0.5 | 15 | ^[22]^ |
| Cu_1_/Ru | 51.27 | 12 | -0.6 | 20 | ^[23]^ |
| Pd_2_Au_1_/RuO_2_ | 75.6 | 37 | -0.5 | 140 | ^[24]^ |
| Zn_1_/In_2_O_3-x_ | 55.8 | 16.5 | -0.7 | 10 | ^[25]^ |
| CuSiO_x_ | 79.01 | / | 0 to -0.2 | 80 | ^[26]^ |
| Ag-CuNi(OH)_2_ | 46.1 | 1 | -0.5 | 50 | ^[27]^ |
| CuWO_4_ | 70.1 | 1 | -0.2 | 10 | ^[28]^ |
| O_L_-Cu | 31.71 | 95 | -0.7 | 32 | ^[29]^ |
| V_o_-S-IO-6 | 60.6 | 1 | -0.6 | 12 | ^[30]^ |
| SrCo_0.39_Ru_0.61_O_3−δ_ | 34.1 | 17 | -0.7 | 360 | ^[31]^ |
| AuCu-RP | 88.5 | 1 | -0.6 | 12 | ^[32]^ |
| a-TiS_2_ | 34.5 | 20 | -0.7 | 50 | ^[33]^ |
| Cu_1_-MoS_2_ | 57.02 | 9 | -0.6 | 20 | ^[34]^ |
| PCOF-34-Fe | 90.0 | 10 | -0.5 | 100 | ^[35]^ |
| CuCo@N-CNT | 73.69 | 70 | -0.8 | 10 | ^[36]^ |
| Ru–Cu_9_Bi/CNT | 75.6 | 3 | -0.4 | 150 | ^[37]^ |
| γ-Fe_2_O_3_@Ni-HITP | 67.2 | 90 | -0.8 | 150 | ^[38]^ |
| PcNi-Fe-O | 54.1 | 10.1 | -0.6 | 20 | ^[39]^ |
| CuPc-Amino | 11.9 | 8.2 | −1.6 | 3.3 | ^[40]^ |
| FeNC-Fe_1_N_4_ | 66.5 | 2 | -0.6 | 200 | ^[41]^ |
| Co NPs@C | 54.3 | 9 | -0.5 | 3 | ^[42]^ |
| TiO_2_-C | 48.88 | 4.5 | -0.9 | 10 | ^[43]^ |
| Cu-N_4_ | 28 | 27 | -0.9 | / | ^[44]^ |
| CN | 62 | 0.56 | −0.5 | 6 | ^[45]^ |
| N-PHCS | 19.1 | 2.5 | -1.0 | 24 | ^[46]^ |
| Zn/Cu | 75 | 1 | -0.6 | 32 | ^[47]^ |
| V_o_-InOOH | 51.0 | 0.6 | -0.5 | 24 | ^[48]^ |
| Mo_2_C/C | 44.80 | 6 | -0.5 | 12 | ^[49]^ |
| H-PdZn | 24.39 | 5 | -0.5 | 30 | ^[50]^ |
| Fe–Ni | 17.8 | 45 | -1.5 | 5.6 | ^[51]^ |
| VB_12_-CNTs | 26.04 | 1.3 | -0.5 | 50 | ^[52]^ |
| Cu_1_Mo_1_/NC | 15.2 | 38 | −1.05 | 10 | ^[53]^ |
| Fe-TPP/CNTs | 27.70 | / | −0.2 to −0.8 | 8 cycles | ^[54]^ |

**Table S3.** Comparison of the long-term stability of catalysts for the electro-synthesis of urea from CO_2_ and NO_3_⁻.


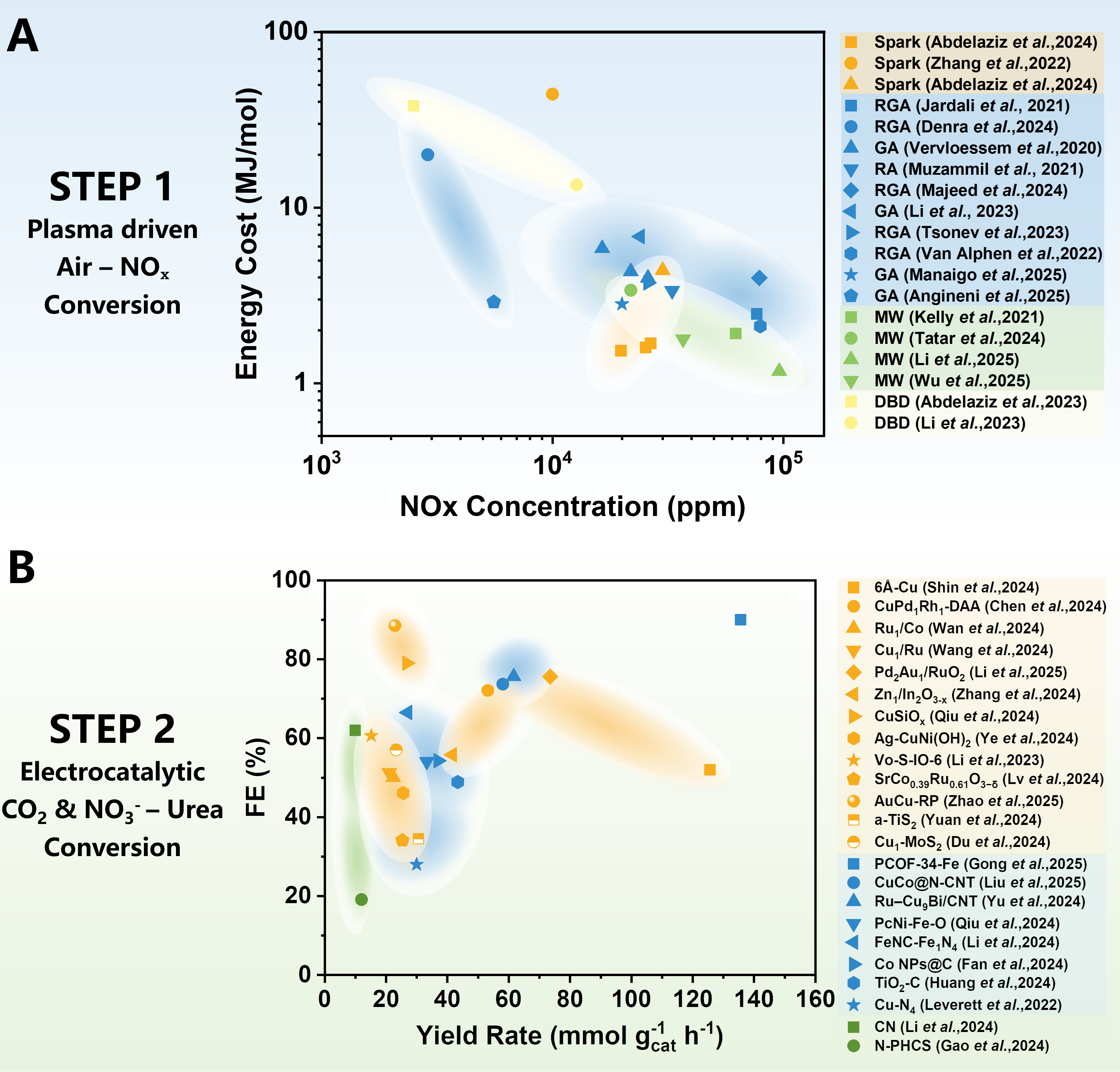


**Figure S1.** (A) Summary of plasma-assisted nitrogen oxidation performance over the last five years (2020-2025) and (B) summary of catalyst performance for nitrate and CO2 urea electrosynthesis over the last three years (2022-2025). The source literature for the data points is organized in SI tables.

**Testing/analysis Methods for Urea**

| Detection method | Qualitative/quantitative | Advantage | Limitations |
| --- | --- | --- | --- |
| UV-Vis spectrophotometry by urease method | Quantitative | High accessibility, facile and easy-to-use detection via UV-vis measurements | Strong interference by NH_4_^+^, NO_2_^−^and metal ions, many influencing factors in enzymes |
| UV-Vis spectrophotometry by diacetylmonoxime method | Quantitative | Low limit of detection (LOD ∼ 0.15 ppm), high accessibility, facile and easy-to-use detection via UV-vis measurements | Strong interference by NO_2_^−^or NH_4_^+^, reducing agents like thiourea and thiosulfate, time-sensitive (heating and cooling time) |
| NMR | Quantitative | High anti-interference ability, ^13^C NMR and^15^N NMR can be used for isotope labeling | Very close position of the urea peak at 5.6 ppm to the water peak at 4.7 ppm affecting the signal-to-noise ratio. Interfering effects of HCO_3_−/CO_3_^2−^, relatively high LOD |
| HPLC/HPLC-MS | Quantitative | High anti-interference ability, isotope labeling possible by MS | Relatively high LOD, separation of side products and urea in column challenging |
| Infrared reflection–absorption spectroscopy(IRRAS) | Qualitative | Detection of adsorbed intermediates selectively due to selection rules (if PM-IRRAS) | Sensitive to matrices |
| Surface-enhanced Raman spectroscopy(SERS) | Semi-quantitative | High sensitivity, selective, identification of adsorbed intermediate due to selection rules | May need immobilization of specific urea-sensing enzymes on the substrate to increase selectivity |
| Attenuated total internal reflectance Fourier transform infrared spectroscopy(ATR-FTIR) | Semi-quantitative | Specification of intermediate functionalities and C–N coupling, sensitivity, compatible with various matrices | Detects product in the liquid interface and not adsorbed ones |

**Table S4.** Testing/analysis methods for urea and their advantages and limitations.

**Energy penalties of pulsed vs. constant potential**

| Catalyst | Electrolysis mode | FE_urea_ (%) | Energy consumption (kWh/kg) | Reduction |
| --- | --- | --- | --- | --- |
| Fe-TPP/CNTs | constant potential (-0.8V) | 19.15 | 75.6 | / |
| Fe-TPP/CNTs | pulsed electrolysis | 27.7 | 44.6 | 41% |
| PdCu | constant potential(-0.6V) | 40.29 | ~33.0 | / |
| PdCu | pulsed electrolysis | 70.36 | 16.5 | >50% |

**Table S5.** Energy penalties of pulsed electrolysis and constant voltage electrolysis modes. Data sourced from^[54]^.

**Reference**

[1] M. Muhyuddin, G. Zuccante, P. Mustarelli, J. Filippi, A. Lavacchi, L. Elbaz, Y.-H. Chen, P. Atanassov, C. Santoro, *Energy & Environmental Science* **2024**, 17, 3739.

[2] A. A. Abdelaziz, Y. Teramoto, D.-Y. Kim, T. Nozaki, H.-H. Kim, *Plasma Chemistry and Plasma Processing* **2024**, 44, 1493.

[3] S. Zhang, L. Zong, X. Zeng, R. Zhou, Y. Liu, C. Zhang, J. Pan, P. J. Cullen, K. Ostrikov, T. Shao, *Green Chemistry* **2022**, 24, 1534.

[4] A. A. Abdelaziz, Y. Teramoto, T. Nozaki, H.-H. Kim, *Chemical Engineering Journal* **2023**, 470, 144182.

[5] F. Jardali, S. Van Alphen, J. Creel, H. Ahmadi Eshtehardi, M. Axelsson, R. Ingels, R. Snyders, A. Bogaerts, *Green Chemistry* **2021**, 23, 1748.

[6] A. Denra, S. Saud, D. B. Nguyen, Q. T. Trinh, T.-K. Nguyen, H. An, N.-T. Nguyen, S. Teke, Y. S. Mok, *Journal of Cleaner Production* **2024**, 436, 140618.

[7] E. Vervloessem, M. Aghaei, F. Jardali, N. Hafezkhiabani, A. Bogaerts, *ACS Sustainable Chemistry & Engineering* **2020**, 8, 9711.

[8] I. Muzammil, D. H. Lee, D. K. Dinh, H. Kang, S. A. Roh, Y.-N. Kim, S. Choi, C. Jung, Y.-H. Song, *RSC Advances* **2021**, 11, 12729.

[9] M. Majeed, M. Iqbal, M. Altin, Y.-N. Kim, D. K. Dinh, C. Lee, Z. Ali, D. H. Lee, *Chemical Engineering Journal* **2024**, 485, 149727.

[10] S. Li, T. van Raak, R. Kriek, G. De Felice, F. Gallucci, *ACS Sustainable Chemistry & Engineering* **2023**, 11, 12821.

[11] I. Tsonev, C. O’Modhrain, A. Bogaerts, Y. Gorbanev, *ACS Sustainable Chemistry & Engineering* **2023**, 11, 1888.

[12] S. Van Alphen, H. Ahmadi Eshtehardi, C. O'Modhrain, J. Bogaerts, H. Van Poyer, J. Creel, M.-P. Delplancke, R. Snyders, A. Bogaerts, *Chemical Engineering Journal* **2022**, 443, 136529.

[13] F. Manaigo, O. Samadi Bahnamiri, A. Chatterjee, A. Panepinto, A. Krumpmann, M. Michiels, A. Bogaerts, R. Snyders, *ACS Sustainable Chemistry & Engineering* **2024**, 12, 5211.

[14] J. Angineni, P. M. K. Reddy, S. Anga, P. V. Somaiah, *Plasma Processes and Polymers* **2025**, 22, 2400209.

[15] S. Kelly, A. Bogaerts, *Joule* **2021**, 5, 3006.

[16] Y. Li, S.-S. Li, K. Lu, Z. Zheng, Y. Feng, D.-Z. Yang, *Journal of Environmental Chemical Engineering* **2025**, 13, 115887.

[17] S. Wu, Y. Liao, K. Feng, F. Zheng, W. Xiao, *Journal of Physics D: Applied Physics* **2025**, 58, 015205.

[18] A. A. Abdelaziz, Y. Teramoto, T. Nozaki, H.-H. Kim, *ACS Sustainable Chemistry & Engineering* **2023**, 11, 4106.

[19] J. Li, C. Lan, L. Nie, D. Liu, X. Lu, *Chemical Engineering Journal* **2023**, 478, 147483.

[20] S. Shin, S. Sultan, Z.-X. Chen, H. Lee, H. Choi, T.-U. Wi, C. Park, T. Kim, C. Lee, J. Jeong, H. Shin, T.-H. Kim, H. Ju, H. C. Yoon, H.-K. Song, H.-W. Lee, M.-J. Cheng, Y. Kwon, *Energy & Environmental Science* **2023**, 16, 2003.

[21] K. Chen, D. Ma, Y. Zhang, F. Wang, X. Yang, X. Wang, H. Zhang, X. Liu, R. Bao, K. Chu, *Advanced Materials* **2024**, 36, 2402160.

[22] Y. Wan, Z. Zhang, X. Wang, X. Yang, H. Zhang, K. Chu, *Advanced Functional Materials* **2024**, 34, 2406438.

[23] F. Wang, S. Shang, Z. Li, Z. Zhang, K. Chu, *ACS Energy Letters* **2024**, 9, 4624.

[24] J.-Y. Li, Y.-F. Li, L.-S. Li, Z. Jiang, Y. Chen, B. Y. Xia, *Angewandte Chemie International Edition* **2025**, 64, e202421266.

[25] Y. Zhang, Z. Li, K. Chen, X. Yang, H. Zhang, X. Liu, K. Chu, *Advanced Energy Materials* **2024**, 14, 2402309.

[26] W. Qiu, S. Qin, Y. Li, N. Cao, W. Cui, Z. Zhang, Z. Zhuang, D. Wang, Y. Zhang, *Angewandte Chemie International Edition* **2024**, 63, e202402684.

[27] W. Ye, Y. Zhang, L. Chen, F. Wu, Y. Yao, W. Wang, G. Zhu, G. Jia, Z. Bai, S. Dou, P. Gao, N. Wang, G. Wang, *Angewandte Chemie International Edition* **2024**, 63, e202410105.

[28] Y. Zhao, Y. Ding, W. Li, C. Liu, Y. Li, Z. Zhao, Y. Shan, F. Li, L. Sun, F. Li, *Nature Communications* **2023**, 14, 4491.

[29] X. Wei, S.-Q. Liu, H. Liu, Y. Ding, P.-X. Lei, S. Wu, L. Song, X.-Z. Fu, J.-L. Luo, *Journal of the American Chemical Society* **2025**, 147, 6049.

[30] Z. Li, P. Zhou, M. Zhou, H. Jiang, H. Li, S. Liu, H. Zhang, S. Yang, Z. Zhang, *Applied Catalysis B: Environmental* **2023**, 338, 122962.

[31] L. Lv, H. Tan, Y. Kong, B. Tang, Q. Ji, Y. Liu, C. Wang, Z. Zhuang, H. Wang, M. Ge, M. Fan, D. Wang, W. Yan, *Angewandte Chemie International Edition* **2024**, 63, e202401943.

[32] C. Zhao, Y. Jin, J. Yuan, Q. Hou, H. Li, X. Yan, H. Ou, G. Yang, *Journal of the American Chemical Society* **2025**, 147, 8871.

[33] D. Yuan, Y. Jiang, F. Wang, D. Ma, K. Chu, *Journal of Colloid and Interface Science* **2025**, 679, 60.

[34] W. Du, Z. Sun, K. Chen, Y. Wei, R. Bao, K. Chu, *Advanced Energy Materials* **2024**, 14, 2401765.

[35] C. Gong, Y. Peng, M. Xu, X. Wei, G. Sheng, J. Liu, X. Wu, X. Han, F. Dai, J. Dong, Z. Chen, Y. Zhu, W. Ye, Y. Cui, *Nature Synthesis* **2025**.

[36] J. Liu, S. Zhang, Z. Mao, W. Li, M. Jin, H. Yin, Y. Zhang, G. Wang, H. Zhang, *Inorganic Chemistry Frontiers* **2025**.

[37] Y. Yu, Y. Sun, J. Han, Y. Guan, H. Li, L. Wang, J. Lai, *Energy & Environmental Science* **2024**, 17, 5183.

[38] D.-S. Huang, X.-F. Qiu, J.-R. Huang, M. Mao, L. Liu, Y. Han, Z.-H. Zhao, P.-Q. Liao, X.-M. Chen, *Nature Synthesis* **2024**, 3, 1404.

[39] X.-F. Qiu, J.-R. Huang, C. Yu, X.-M. Chen, P.-Q. Liao, *Angewandte Chemie International Edition* **2024**, 63, e202410625.

[40] H. Li, L. Xu, S. Bo, Y. Wang, H. Xu, C. Chen, R. Miao, D. Chen, K. Zhang, Q. Liu, J. Shen, H. Shao, J. Jia, S. Wang, *Nature Communications* **2024**, 15, 8858.

[41] Z. Li, M. Xu, J. Wang, Y. Zhang, W. Liu, X. Gu, Z.-K. Han, W. Ye, G. Li, *Small* **2024**, 20, 2400036.

[42] X. Fan, C. Liu, X. He, Z. Li, L. Yue, W. Zhao, J. Li, Y. Wang, T. Li, Y. Luo, D. Zheng, S. Sun, Q. Liu, L. Li, W. Chu, F. Gong, B. Tang, Y. Yao, X. Sun, *Advanced Materials* **2024**, 36, 2401221.

[43] X. Huang, Y. Li, S. Xie, Q. Zhao, B. Zhang, Z. Zhang, H. Sheng, J. Zhao, *Angewandte Chemie International Edition* **2024**, 63, e202403980.

[44] J. Leverett, T. Tran-Phu, J. A. Yuwono, P. Kumar, C. Kim, Q. Zhai, C. Han, J. Qu, J. Cairney, A. N. Simonov, R. K. Hocking, L. Dai, R. Daiyan, R. Amal, *Advanced Energy Materials* **2022**, 12, 2201500.

[45] Y. Li, S. Zheng, H. Liu, Q. Xiong, H. Yi, H. Yang, Z. Mei, Q. Zhao, Z.-W. Yin, M. Huang, Y. Lin, W. Lai, S.-X. Dou, F. Pan, S. Li, *Nature Communications* **2024**, 15, 176.

[46] W. Gao, Q. Wu, X. Fan, N. Lu, Y. Liu, X. Quan, *ACS Applied Materials & Interfaces* **2024**, 16, 50726.

[47] Y. Luo, K. Xie, P. Ou, C. Lavallais, T. Peng, Z. Chen, Z. Zhang, N. Wang, X.-Y. Li, I. Grigioni, B. Liu, D. Sinton, J. B. Dunn, E. H. Sargent, *Nature Catalysis* **2023**, 6, 939.

[48] C. Lv, C. Lee, L. Zhong, H. Liu, J. Liu, L. Yang, C. Yan, W. Yu, H. H. Hng, Z. Qi, L. Song, S. Li, K. P. Loh, Q. Yan, G. Yu, *ACS Nano* **2022**, 16, 8213.

[49] Y. Zhang, X. Fan, X. He, T. Yan, Y. Yao, D. Zheng, J. Zhao, Q. Cai, Q. Liu, L. Li, W. Chu, S. Sun, X. Sun, *Chinese Chemical Letters* **2024**, 35, 109806.

[50] Z. Wang, Y. Wang, S. Xu, K. Deng, H. Yu, Y. Xu, H. Wang, L. Wang, *Journal of Materials Chemistry A* **2025**, 13, 305.

[51] X. Zhang, X. Zhu, S. Bo, C. Chen, M. Qiu, X. Wei, N. He, C. Xie, W. Chen, J. Zheng, P. Chen, S. P. Jiang, Y. Li, Q. Liu, S. Wang, *Nature Communications* **2022**, 13, 5337.

[52] M. Cong, Q. Liu, D. Wang, S. Hao, Z. Han, H. Xu, M. Guo, X. Ding, Y. Gao, *Applied Catalysis B: Environment and Energy* **2024**, 351, 123941.

[53] H. Wang, S. Man, H. Wang, V. Presser, Q. Yan, *Chemical Engineering Journal* **2024**, 497, 154455.

[54] Q. Hu, W. Zhou, S. Qi, Q. Huo, X. Li, M. Lv, X. Chen, C. Feng, J. Yu, X. Chai, H. Yang, C. He, *Nature Sustainability* **2024**, 7, 442.
